# A Structured Analytical Framework to Facilitate EU Food Exports to the USA: A Case Study Analyzing Barriers and Support Strategies

**DOI:** 10.3390/foods15040761

**Published:** 2026-02-19

**Authors:** Andrea Gori, Valentina Garretto, Paola Vannucci, Gaetano Liuzzo, Giovanni Munaò, Lara Tinacci, Roberta Nuvoloni, Andrea Armani

**Affiliations:** 1 Department of Veterinary Sciences, University of Pisa, Viale delle Piagge 2, 56124 Pisa, Italy; v.garretto@studenti.unipi.it (V.G.); lara.tinacci@unipi.it (L.T.); roberta.nuvoloni@unipi.it (R.N.); 2 Azienda USL Toscana Centro, Via Salvanti, Calenzano, 50041 Florence, Italy; paola.vannucci@uslcentro.toscana.it (P.V.); giovanni.munao@uslcentro.toscana.it (G.M.); 3 Centro Servizi AUSL Modena, Strada Martiniana 21, Baggiovara, 41126 Modena, Italy; g.liuzzo@ausl.mo.it

**Keywords:** FSIS, SSOPs, HACCP system, pre-shipment review, ministerial circulars

## Abstract

Exporting food products from the European Union (EU) to the United States of America (USA) involves navigating complex regulations and procedural barriers that hinder market access. Italian food businesses (FBs), particularly small and medium-sized enterprises (SMEs), often face difficulties accessing clear guidance, as national procedures are scattered across multiple sources. This paper proposes a structured three-step analytical framework to support EU FBs: product-specific analysis, identification of relevant EU and USA legislation, comparative legislative analysis via concordance tables, and identification of procedures to integrate into the Food Safety Management System. The framework was applied to an Italian medium-sized FB exporting pork-based pasta sauce to the USA. Beyond the specific case study, the proposed analytical framework was designed to be transferable and adaptable to other food categories and destination markets, providing a structured methodological tool to support regulatory alignment. In this sense, the framework can be considered product-independent but process-specific. As such, it can support both FBs and Competent Authorities in conducting risk-based assessments of regulatory equivalence and export compliance. Results indicated the need for Sanitation Standard Operating Procedures (SSOPs), thermal process validation, direct verification activities, and pre-shipment review. Findings emphasize that operational and procedural barriers disproportionately affect SMEs, highlighting the importance of targeted support to facilitate market access and strengthen certification systems.

## 1. Introduction

In an increasingly globalized food system, the export of Italian agri-food products outside the European Union (EU) plays a strategic role not only in terms of economic and reputational value, but also in shaping the interaction between national food safety systems and international regulatory frameworks [1,2,3]. Over the years, Italy has progressively consolidated its presence in the global agri-food market, achieving a relevant growth in exports [4]. From 2010 to 2022, there was a steady increase in the value of exported products, with only minimal exceptions [5]. In the second quarter of 2024, agri-food exports increased further, reaching €16.8 billion, representing a growth of 8.2% compared to the same period of the previous year [6]. Despite notable growth in markets such as Australia (+18%) and Japan, where the export value increased by nearly 50% following a slowdown in 2023, these countries remain far from matching the United States of America (USA) as a key destination for Italian agri-food products [7]. Indeed, the USA recorded a +17% increase in imports of Italian agri-food products in the first half of 2024 compared to the same period in 2023 [7].

Exporting food products is a complex activity that requires compliance with numerous guarantees [8,9,10,11]. Italian food businesses (FBs), being based in the EU, must comply with the safety and hygiene requirements set forth in Regulations (EC) No 852/2004 and No 853/2004 and subsequent amendments and supplements [12,13]. Article 4 of Regulation (EC) No 852/2004 requires compliance with general hygiene requirements, which are supplemented by specific hygiene requirements for food of animal origin [12]. These requirements correspond to what are internationally known as the so-called Prerequisite Programs (PRPs), as defined by the World Health Organization (WHO), the Food and Agriculture Organization of the United Nations (FAO), the Codex Alimentarius, and the International Organization for Standardization (ISO). Additionally, Article 5 of Regulation (EC) No 852/2004 requires FBs to establish, implement, and maintain procedures based on the principles of the Hazard Analysis and Critical Control Points (HACCP) system. The HACCP system is internationally recognized as an essential tool for enabling FBs to identify and manage food-related hazards [14]. Together with the principles set out in Regulation (EC) No 178/2002 and subsequent amendments and supplements [15], including risk analysis, the precautionary principle, transparency, the primary responsibility of FBs, and traceability, these form the foundation of the EU Food Safety Management System (FSMS), which all FBs must comply with [16]. In the case of exports, beyond compliance with EU FSMS, FBs must also ensure that their products do not pose risks to animal or plant populations in the destination country [17], while complying with that country’s specific safety and hygiene standards [18]. These requirements are defined through negotiations between: (i) the Competent Authority (CA) of the exporting and importing countries, or (ii) the European Commission (EC) and the CA of the importing country [15]. In both cases, the process results in an international agreement which, when managed by the EC, applies to all Member States [19]. Such agreements are based on internationally recognized standards, including the food safety standards of the Codex Alimentarius, the animal health standards set out in the Terrestrial Animal Health Code of the World Organization for Animal Health (WOAH), and the provisions of the World Trade Organization’s (WTO) Sanitary and Phytosanitary (SPS) Agreement [20]. Although the responsibility for demonstrating equivalence lies with the exporting country, international standards require countries to recognize that different inspection and certification systems can achieve the same level of safety and, therefore, be considered equivalent [21,22,23,24]. Compliance with the agreement’s requirements is ensured by a health certificate issued by the Member State’s CA, which must accompany the exported food [17]. The health certificate specifies the conditions the FB must meet to export to the destination country, and the CA is responsible for verifying compliance prior to its issuance. However, in many cases, FBs must first be included in lists of authorized establishments, managed either by the importing country’s authority or by that of the Member State. Such inclusion, generally subject to the verification of specific requirements by the CA, is a mandatory step before initiating the health certification procedure [8,18].

**Figure 1:**
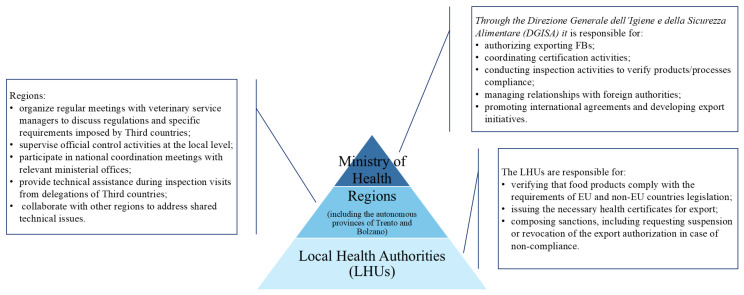
Role of the Italian Competent Authorities in the field of export activities. FBs, food businesses; EU, European Union.

In Italy, CAs operate within a system of multilevel governance: central, regional, and local [25]. Each level plays a key role in the field of export activities [18,26,27] as illustrated in Figure 1. Within this framework, the Ministry of Health also contributes by issuing internal provisions, typically in the form of circulars. Ministerial circulars represent an essential tool for the comprehension and application of legislation, providing instructions and clarifications on the procedures to be followed. These circulars provide guidance to Local Health Units (LHUs) on the interpretation and enforcement of regulations [28]. In the case of export, circulars provide instructions and clarifications to ensure that official veterinarians of LHUs responsible for export certification procedures are properly informed on the verifications they are required to perform in accordance with the specific conditions set by the destination country. Although primarily addressed to CAs, these documents are also transmitted, for knowledge, to trade associations, which in turn make them available to FBs. Indeed, FBs can refer to these documents as guidance to meet the general and specific requirements established by the destination country. However, the procedures that Italian FBs are required to implement for export are not currently consolidated in a single manual or set of guidelines. As a result, FBs must navigate the official website of the Ministry of Health on their own or rely on documents that may be provided by trade associations. This fragmentation may largely stem from the layering of export-specific requirements and compliance obligations that have developed over time. As a result, export-relevant regulatory information has expanded in volume without being fully integrated into a single, operationally accessible system. This fragmentation of information can lead to uncertainty, especially when FBs are faced with interpreting ministerial circulars, which, being primarily intended for the CA, are often highly technical and complex. In addition, health certificates do not always contain sufficiently detailed instructions that effectively guide FBs in implementing the required measures (authors’ note). The situation becomes even more complex when considering that such requirements vary not only from one country to another but also depending on the type of product being exported, making compliance a frequently challenging and burdensome task (authors’ note). In this context, especially small and medium-sized enterprises (SMEs) may struggle to adapt their facilities and production processes to meet export requirements, also due to the high costs that such adjustments may involve [29].

Based on these premises, the overall aim of this study, conducted in collaboration with the LHU “Azienda USL Toscana Centro”, was to apply a structured and transferable analytical framework to systematically analyze regulatory requirements governing the export of Italian food products to third countries, using the USA as a case study. The specific objectives were: (i) to identify and analyze the legally binding regulatory, administrative, and procedural requirements for export authorization to the USA; (ii) to assess the concrete adjustments required within the FSMS of an Italian medium-sized FB producing pork-based pasta sauces to achieve compliance; and (iii) to identify operational and procedural barriers that may hinder market access, particularly for SMEs. By addressing these objectives, the study wanted to contribute to the practical understanding of regulatory equivalence and to provide policy- and practice-relevant insights to support FBs and CAs in managing export certification processes more effectively, while offering a framework that can be applied beyond the USA context.

## 2. Materials and Methods

### 2.1. Schematic Overview of the Analytical Framework

**Figure 2:**
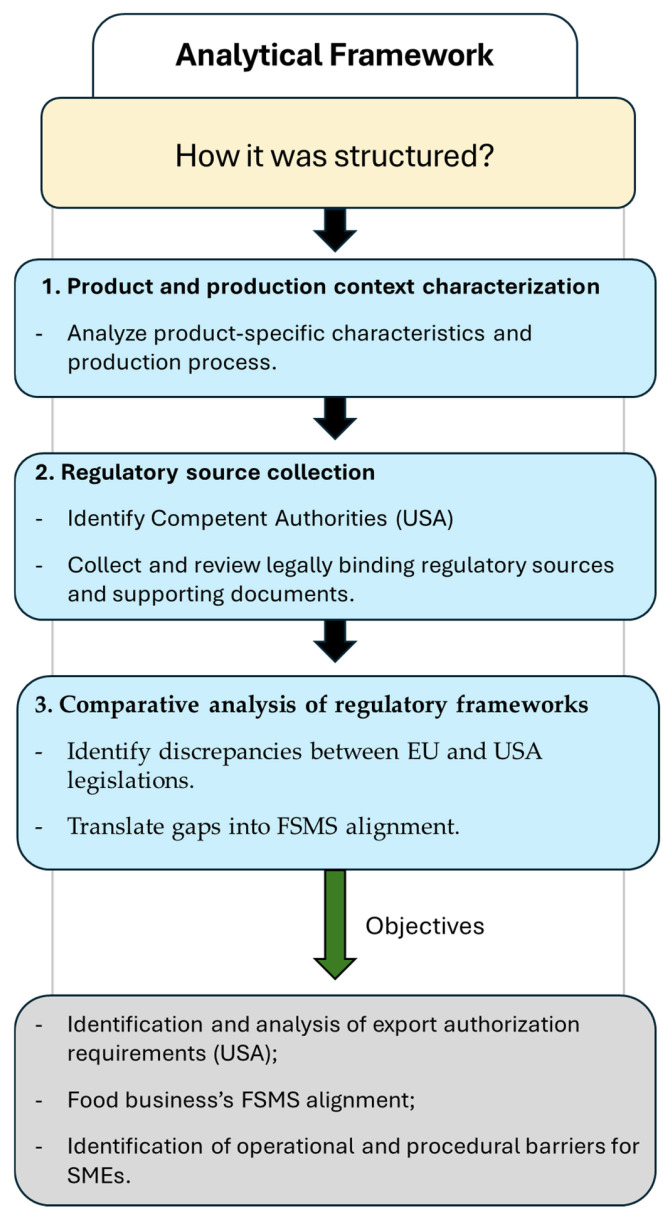
Schematic flow diagram summarizing the analytical framework applied in this study. USA, United States of America; EU, European Union; FSMS, Food Safety Management System; SMEs, small and medium-sized enterprises.

Figure 2 presents a schematic flow diagram summarizing the analytical framework applied in this study and outlining the sequential steps that structure the entire Materials and Methods Section. The framework was developed as an operational tool to address the overall aim of the study and was applied through three main analytical phases: (i) characterization of the product and production context, (ii) identification of the CAs and collection of legally binding EU and USA regulatory sources, and (iii) comparative analysis of the two regulatory frameworks. The flow diagram also reflects the specific objectives of the study.

### 2.2. Analysis of the Production Plant and the Product to Be Exported

From March to May 2023, several visits were conducted, together with the official veterinarian responsible for export certification procedures of the LHU “Azienda USL Toscana Centro”, to an FB approved under Regulation (EC) No 853/2004 for the production of canned foods. The study period reflects the regulatory assessment rather than a longitudinal production analysis. Being “approved” means that the FB fully complies with all relevant EU hygiene and safety requirements, including those specific to the production of canned foods. The case study originated from the FB’s expressed interest in initiating export activities towards the USA, which led the CA to carry out a preliminary assessment of the production plant and products. The FB was selected as an information-rich case, providing a real-world context to apply the analytical framework to a medium-sized FB facing concrete regulatory challenges for exporting to the USA. This choice allowed the study to capture both operational and procedural adaptations required within the FSMS, while maintaining transparency about the practical constraints encountered by SMEs. In the context of supporting the FB’s compliance process for access to the USA market, the CA requested scientific and technical assistance from the Department of Veterinary Sciences of the University of Pisa.

At that time, the FB was managed by approximately 50 employees with an annual revenue of around 20 million euros. Therefore, the FB fell within the definition of a medium-sized enterprise according to European Commission Recommendation 2003/361/EC [30]. The FB was equipped with two distinct and physically separated production lines: one dedicated to sauces and the other to dry products. Specifically, the line dedicated to sauces included the production of pasta sauces made with meat (pork, beef, and game), fish, bivalve mollusks and crustaceans, dairy products, as well as various vegetable-based sauces. In parallel, the other line was dedicated to the production and packaging of dried vegetable products.

In response to FB’s request to export meat-based pasta sauces to the USA, the line dedicated to pork-based pasta sauce was identified by the official veterinarian as among the meat-based products processed at the FB, only pork-based pasta sauce met the eligibility criteria for export to the USA [31]. This eligibility depends on specific requirements established under the bilateral agreement between Italy and the USA for meat products, including the assessment of the health status of animals in the exporting country or region with respect to notifiable diseases. In fact, the Animal and Plant Health Inspection Service (APHIS) restricts the importation of certain animal products into the USA based on the health status of the exporting or transit country or region. At the time of the study, only pork-based products met these requirements, while other meat categories processed at the plant, such as beef, game, or poultry, were not eligible for export to the USA due to non-compliance with the health status of the Italian territory.

### 2.3. Regulatory Source Collection

**Figure 3:**
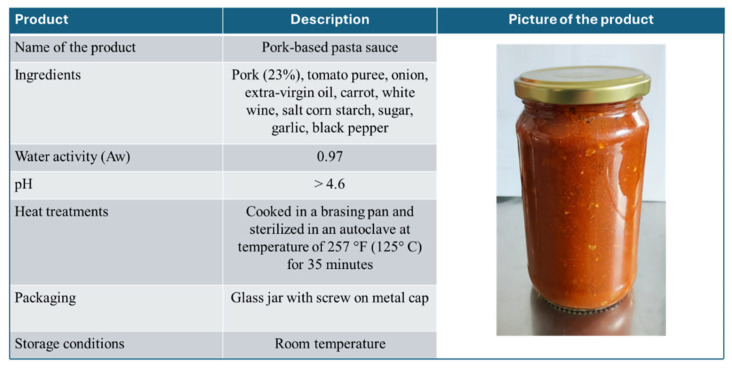
Analysis of product characteristics intended for export to the United States of America.

To determine the relevant regulatory framework, it was necessary to define the classification of the products according to the USA legislation. A summary of the main characteristics of the product intended for export to the USA is provided in Figure 3. Based on these characteristics, the product under study was classified as a “Thermally processed and commercially sterile” (TPCS) meat-based product. Therefore, the Code of Federal Regulations (CFR) and the Food Safety and Inspection Service (FSIS) database were consulted. Specifically, searches were conducted on the FSIS website, as well as through search engines using relevant keywords (USA food legislation, USA food safety, Code of Federal Regulation, United States Department of Agriculture (USDA), FSIS-USDA, FSIS canned food, thermally processed commercially sterile products), to identify the mandatory legislation, guidelines, and other supplementary documents related to both general and specific requirements for the product under study.

As regards to the EU legislation, relevant legislative sources concerning general and specific food hygiene and safety requirements were identified through searches on the official EU website (eur-lex.europa.eu) and through targeted search engine queries using keywords (EU food legislation, EU food safety, EU canned food, HACCP EU, EU water quality directive, EU animal by-products regulation). In addition, EU guidance documents were also investigated to ensure a comprehensive understanding of the applicable framework. Finally, ministerial circulars related to the USA export requirements were collected. All legislative sources collected refer to the current consolidated versions, which consider all amendments and supplements made over time, to ensure an up-to-date analysis in line with the applicable regulatory framework.

The regulatory source collection focused exclusively on mandatory and legally binding legislative instruments, including regulations, official guidelines, and ministerial circulars applicable to export authorization. Voluntary private certification schemes were not included in the comparative analysis, as they do not constitute legal requirements for export authorization under FSIS jurisdiction.

### 2.4. Comparative Analysis and FSMS Alignment

**Figure 4:**
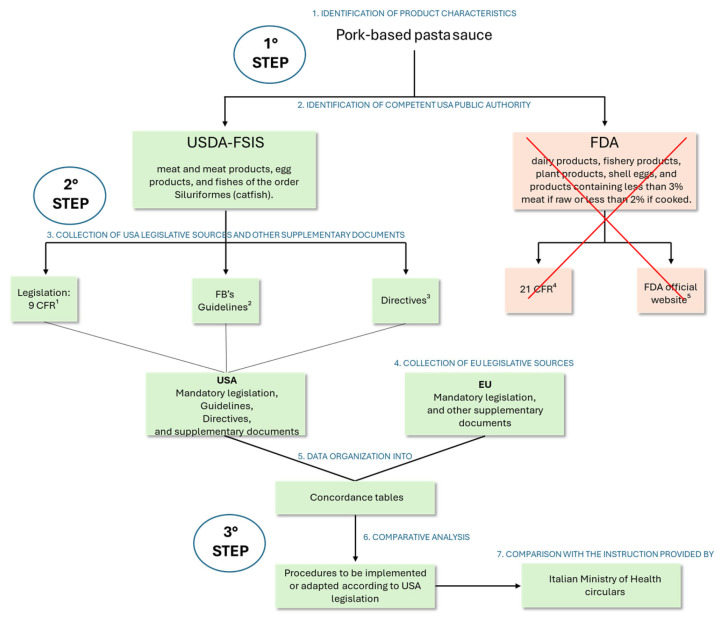
Schematic representation illustrating the application of the analytical framework to the United States of America case study. The circles indicate the three main steps of the general framework (Figure 2), operationally implemented here through the specific activities carried out in this case. FSIS, Food Safety and Inspection Service; FDA, Food and Drug Administration; CFR, Code of Federal Regulation; FB, food business; USDA, United States Department of Agriculture; EU, European Union; USA, United States of America. ^1^ https://www.ecfr.gov/current/title-9 (accessed on 20 February 2025). The red cross highlights the exclusion of the FDA jurisdiction and related legal sources, as the case study concerns a product falling under USDA-FSIS authority. ^2^ https://www.fsis.usda.gov/policy/fsis-guidelines (accessed on 20 February 2025). ^3^ https://www.fsis.usda.gov/policy/directives-notices-guidelines/fsis-directives (accessed on 20 February 2025). ^4^ https://www.ecfr.gov/current/title-21 (accessed on 20 February 2025). ^5^ https://www.fda.gov/ (accessed on 20 February 2025).

Firstly, general (PRPs) and specific (HACCP) requirements were identified from the collected legislative sources (both the USA and the EU). These were then supplemented with the relevant guidance documents covering the different areas of interest. The information was organized into concordance tables, and the tabulated data was compared to highlight differences representing the procedures that the FB must implement in its EU FSMS to comply with USA legislation. The identification and comparison process followed a stepwise logic: regulatory sources were first screened to extract relevant general and product-specific requirements; these requirements were then mapped against the corresponding EU FSMS elements to highlight gaps or necessary adaptations. Finally, the identified procedures to be implemented were compared with the instructions provided by the pertinent ministerial circulars. In this context, Figure 2 provides a schematic overview of the general analytical framework, while Figure 4 illustrates its operational application to the USA case study, detailing how regulatory sources, guidance documents, and institutional responsibilities were concretely analyzed and integrated to identify FSMS adaptations. Together, the two figures describe different levels of the same methodological approach, moving from a transferable analytical structure to its case-specific implementation. To enhance methodological rigor and reduce the risk of researcher bias, the document collection (USA and EU mandatory legislation, guidelines, and other supplementary documents), data tabulation, and identification of procedures to be implemented were carried out jointly by two researchers. A third researcher then conducted a quality control stage, verifying that all relevant documents had been collected and carefully re-reading them to identify any missing sources or overlooked elements. Therefore, this process incorporated data triangulation by involving multiple sources and perspectives. All materials, including the concordance tables, were then submitted to the official veterinarian, who conducted an independent review of both the collected documentation and the procedures identified, minimizing interpretive bias and increasing methodological robustness. Throughout the process, multiple meetings were held among the team members to iteratively review and validate the findings, ensuring a consistent methodological approach. While the framework is illustrated using a pork-based pasta sauce case for the USA, its structured, stepwise approach is methodological and not product-specific. The same analytical sequence can be applied to other food categories or different third-country markets, requiring only adaptation of the inputs (e.g., regulatory sources and product-specific hazards), while maintaining the integrity of the three-step logic.

## 3. Results and Discussions

### 3.1. USA and EU Legislation Collection and Analysis

#### 3.1.1. USA Mandatory Legislation, Guidelines, and Other Supplementary Documents

In the USA, the main public authorities responsible for ensuring food safety and hygiene are: (i) the Food and Drug Administration (FDA), operating under the Department of Health and Human Services (DHHS), and (ii) the Food Safety and Inspection Service (FSIS), which, together with the APHIS operates within the United States Department of Agriculture (USDA) [31,32,33]. The FDA is responsible for dairy and fishery products, plant-based products, shell eggs, and products containing less than 3% meat if raw or less than 2% if cooked [34]. FSIS, on the other hand, is responsible for meat and meat products, egg products, and fish of the order Siluriformes [11,35,36]. Based on this regulatory framework, it was determined that the product described in Section 2.2 falls under the jurisdiction of FSIS.

Our analysis identified specific regulations addressing both general and specific requirements applicable to food products under FSIS jurisdiction, as outlined in Chapter III of Title 9 of the CFR, which relates to animals and animal products. Within this chapter, Subchapter E outlines the regulatory requirements for the inspection of meat, poultry, and egg products. As regards FSIS guidelines, they cover a variety of topics aimed at helping FBs comply with federal regulations [37]. The collected guidelines provide guidance on maintaining adequate hygiene conditions, developing and implementing Sanitation Standard Operating Procedures (SSOPs), applying HACCP principles, and controlling hazards in meat and poultry establishments.

Similarly, several FSIS directives were collected, which differ in both meaning and structure from European directives. In the USA, these documents provide detailed instructions to the federal CA, referred to as Inspection Program Personnel (IPP), on how to comply with food safety regulations and policies. They cover a wide range of topics, including inspection procedures, enforcement actions, and the handling of adulterated or misbranded products [37]. Although primarily addressed to IPP, the content of these directives may also be useful for FBs. The complete list of USA mandatory legislation, guidelines, directives, and other supplementary documents considered is reported in Table 1.

#### 3.1.2. EU Mandatory Legislation, Guidelines, and Other Supplementary Documents

With regard to the EU, legislative sources concerning both general and specific food hygiene and safety requirements were considered. General hygiene requirements, including PRPs, as well as specific hygiene rules for food of animal origin, were collected and analyzed. Additionally, relevant legislation on the quality of water intended for human consumption, microbiological criteria for foodstuffs, and health rules applicable to animal by-products and derived products not intended for human consumption were reviewed. Regarding requirements for procedures based on HACCP principles, legislation governing these procedures was considered. Furthermore, regulations establishing the regulatory framework for CAs were also examined. Although these latter are primarily addressed to CAs, they can also provide useful guidance for FBs to ensure compliance with EU legislation.

During the analysis of EU legislative sources, similarly to the USA context, it was necessary to consider additional documents beyond binding regulations. For this reason, guidance documents on the implementation of FSMS, with particular focus on good hygiene practices (GHP) and HACCP-based procedures, were examined. Additionally, scientific opinions regarding biological hazards in food were also consulted. The complete list of EU mandatory legislation, guidelines, and other supplementary documents considered is reported in Table 1.

### 3.2. Ministerial Circulars Collection and Analysis

As instruments issued by the Italian Ministry of Health, ministerial circulars are positioned at a different level compared to the USA and the EU regulations. They constitute internal administrative provisions aimed at ensuring uniform action by public authorities [38]. While circulars are not sources of law, they play a fundamental role in the comprehension and application of legislation, serving as reference documents for CAs [27]. Indeed, circulars can serve various functions: some have a regulatory content, outlining procedures and criteria for administrative activities; others are interpretive, providing clarifications on the application of legal provisions; still others are informational, disseminating relevant information for administrative personnel [39]. The ministerial circulars we analyzed perform a dual function, both informational and interpretive, providing practical indications on the application of regulations and clarifying the meaning of certain requirements set by them [39]. As previously mentioned, although these documents are addressed to the CAs, they are also transmitted, for knowledge, to trade associations, which in turn make them available to FBs in the sector. This enables FBs to make use of information originally intended for CAs and adapt it to their operational needs. In this way, the circulars issued by the Italian Ministry of Health support FBs in aligning with current export requirements, thereby exerting an indirect external effect [40].

The Italian Ministry of Health, acting as the CA at the central level, has issued several ministerial circulars aimed at clarifying the implementation of export requirements to the USA and ensuring their uniform application. These circulars, specifically addressing the requirements for inclusion in the USA list of authorized establishments, were therefore collected and analyzed. One ministerial circular provides clear guidance on how to identify the applicable USA regulations based on the product category, thereby enabling FBs to correctly navigate the regulatory framework [41]. Another focuses on official controls for establishments authorized to export food products to the USA [42]. Its annex can also serve as an operational guide for FBs, providing a translation of relevant USA legislation (9 CFR 416 [43] and 9 CFR 417 [44]) along with interpretative clarifications, practical examples, and compliance criteria [42]. A further circular addresses the USA regulation 9 CFR 431 concerning standards for canned products [45]. Furthermore, another ministerial circular provides operational guidance for the application process to be included in the list of establishments authorized to export food products to the USA [46]. Finally, additional ministerial circulars were collected and analyzed to define the measures required to obtain the relevant health certificate (Table 2).

### 3.3. Procedures to Be Implemented or Adapted for Inclusion in the USA List of Authorized Establishments

The comparative analysis of USA and EU legislation, conducted through concordance tables (Table S1), made it possible to highlight the main gaps between the two regulatory systems and to identify the procedures to be implemented in order to align with USA legislation.

No substantial differences were found regarding the PRPs, except for a different naming and categorization of byproducts. In fact, the definition of “animal by-products”, set out in Regulation (EC) No 1069/2009 and subsequent amendments and supplements [47], is not directly comparable to that adopted in USA legislation (9 CFR 301.2), as in the USA, products intended for human consumption are also included within this category. By contrast, the EU definition of “animal by-products” is more closely aligned with the concept of “inedible products”, defined as “adulterated, uninspected, or not intended for use as human food” [48]. Furthermore, unlike the EU regulatory framework, which classifies animal by-products according to specific risk levels (category 1, 2, and 3), the USA legislation does not adopt the same categorization but distinguishes them between “condemned” and “other inedible products” [49]. However, despite these differences, the management of animal by-products in accordance with Regulation (EC) No 1069/2009 is considered to meet the standards set by USA legislation, as both regulatory frameworks aim to ensure the safe handling, processing, and disposal of animal by-products.

By contrast, our analysis showed that the FB is required to implement new procedures and to adapt some of the existing ones. The new procedures to be implemented concern cleaning and sanitization procedures (SSOPs), while the procedures to be adapted relate to the HACCP system.

At the time of this study, the FB had not yet initiated the implementation of the above-mentioned procedures. Therefore, the procedures and recommendations described in the following sections should be regarded as guidelines or suggested steps provided by the CA, to be followed by the FB to enable its inclusion in the list of establishments authorized to export to the USA.

### 3.4. SSOPs

Concerning cleaning and sanitization procedures, the FB under study is required to incorporate SSOPs into its FSMS, as prescribed by 9 CFR 416. SSOPs are written procedures developed and implemented by FBs to prevent direct contamination or adulteration of products and to ensure compliance with quality and safety standards [50,51,52]. These procedures, which are essential for the effective implementation of HACCP plans, specify what must be cleaned and how often, and include monitoring activities, verification, and corrective actions in case of non-compliance [8,51,53,54]. SSOPs are an integral element of the overall daily food handling or processing operation [55]. According to USA legislation (9 CFR 416.11), all FBs must develop, implement, and maintain written SSOPs to prevent both direct and indirect contamination of products. Therefore, FBs wishing to export to the USA, including the one examined in this study, must apply these procedures. SSOPs must be documented in either paper or electronic format and implemented regularly and effectively [8,54]. These procedures must be applied systematically and uniformly along the entire production line. According to the USA requirement, the FB was requested to develop SSOPs divided into pre-operational procedures (to be conducted before product processing) and operational procedures (to be conducted during product processing), both focusing on the effectiveness of cleaning and sanitizing surfaces with direct, indirect, or no contact with food. In addition, the FB is required to produce detailed daily records documenting the proper implementation of SSOPs. These records should include the date, start and end times, compliance status, any corrective actions taken, and the signature or initials of the trained individual responsible for performing the task [56]. Corrective actions must address the handling of potentially contaminated products (if non-compliances occur during operational activities), which are defined under USA legislation as adulterated products. They must also include the restoration of hygienic conditions and the implementation of preventive measures to avoid recurrence. The FB was also informed that records must be retained for at least six months and kept on-site for 48 h after the procedure. After this period, records may be archived off-site but must be made available to FSIS within 24 h upon request in accordance with 9 CFR 416.16 (c). In the USA, SSOPs therefore represent a mandatory requirement for FBs, which are required to develop, implement, and maintain written procedures to ensure food safety and prevent contamination. By contrast, SSOPs are neither formally required nor explicitly mentioned in EU legislation and are therefore not adopted as specific tools within EU FSMSs. Nevertheless, under Regulations (EC) No 852/2004 and No 853/2004, EU FBs must ensure the effectiveness of cleaning and sanitization procedures through the implementation of a verification plan tailored to the type of processing and the intensity of production. Such a plan may include visual inspections, microbiological sampling, and ATP bioluminescence testing of surfaces, equipment, and working environments [53,57]. The frequency of sampling and analysis must be justified based on the FB’s historical data, thereby allowing flexibility in the design and application of verification activities [57]. As a result, the implementation of these procedures in the EU is generally less prescriptive compared to the SSOPs defined in 9 CFR Part 416, which represent a more formalized and standardized approach to the same underlying concept. This implies that EU FBs intending to export to the USA, including the one examined in this study, are required to implement SSOPs as prescribed by USA legislation, as the EU system is not considered fully equivalent to the USA system with regard to this specific aspect. This finding highlights that the requirement to implement SSOPs does not arise from a lack of safety within the EU system, but rather from the absence of formal equivalence recognition by the USA. Consequently, this requirement entails a relevant commitment involving both the direct responsibility of FBs, which must develop, implement, and document their own SSOPs, and the role of the CA, which is responsible for verifying their effective application. In this context, FSIS has developed a model intended to serve as a reference for FBs when drafting their own SSOPs [58]. The FB examined in this study was therefore advised by the CA to use this model as a basis for defining its own procedures.

### 3.5. HACCP System

The HACCP system is globally recognized as the foundation of effective food safety management in the food industry [59]. It is based on a structured approach consisting of seven principles aimed at identifying, evaluating, and controlling food safety hazards, ensuring that such hazards are prevented, eliminated, or reduced to acceptable levels before the product reaches consumers [60,61]. These principles consist of: (1) hazard analysis; (2) identification of critical control points (CCPs); (3) establishment of critical limits; (4) monitoring procedures; (5) corrective actions; (6) verification procedures; and (7) recordkeeping. They are based on the internationally recognized code of practice established by Codex Alimentarius, which serves as a common reference framework for both the USA and EU [14]. To facilitate their application, voluntary third-party certification schemes recognized by the Global Food Safety Initiative, such as FSSC 22000 [62], may support internal system organization and facilitate dialogue with commercial partners. However, these schemes do not substitute official regulatory authorization or equivalence recognition by CAs and therefore play a complementary rather than determinative role in the export process [63].

Despite the existence of a common theoretical framework, our study revealed that some operational and procedural methods for applying these principles differ. According to 9 CFR 417.2(b)(1), FBs are recommended to preliminarily classify their products into one of the nine specific food processing categories as an integral part of HACCP plan implementation (Table S2). This classification represents a step to ensure that food safety hazards are consistently addressed within the appropriate processing context [64]. Based on our analysis of the product characteristics (Figure 3), it was classified as a TPCS product in accordance with 9 CFR 431 [65]. Once classified, the FSIS directly provides a predefined HACCP model for each of the nine food processing categories (Table S2), which FBs can use as a reference, although its adoption is not mandatory. Regarding TPCS products, the reference document is FSIS-GD-2021-0010 [66]. This document provides a concrete example of applying HACCP principles using a representative product for this category. Based on this product, FSIS developed a detailed description of it, a list of the ingredients, a flow diagram of the production process, and a sample hazard analysis. The EU approach is different from the one adopted at the USA level. In fact, there is no EU regulatory provision that foresees specific food processing categories, nor is there a predefined HACCP model for such categories. Thus, it is left to FBs to develop their own HACCP plan, which can make use of sectoral guidelines or general reference documents provided by CAs. The adoption of the USA model, therefore, represents a pragmatic solution to comply with USA requirements. For this reason, the FB was advised by the CA to adopt the USA model to implement its HACCP plan.

#### 3.5.1. Areas Not Requiring Implementation: Hazard Analysis (Principle 1), Identification of CCPs (Principle 2), Critical Limits (Principle 3), Monitoring Procedures (Principle 4), and Corrective Actions (Principle 5)

Regarding hazard analysis (Principle 1) and, in particular, microbiological hazards, the FSIS guideline “FSIS-GD-2021-0010” emphasizes a key concept outlined in 9 CFR 417.2(b)(3). According to this provision, “HACCP plans for thermally processed commercially sterile products do not have to address the food safety hazards associated with microbiological contamination if the product is produced in accordance with the requirements of part 431”. Nevertheless, this does not preclude the FB from voluntarily including the analysis of microbiological hazards within the HACCP plan. In any case, FSIS provides an additional guideline concerning the microbiological aspects of TPCS and shelf-stable meat and poultry products [67]. This document provides useful details for identifying relevant pathogens in TPCS products, distinguishing them from spoilage microorganisms, and considering differences between low-acid products and low-acid acidified products. Low-acid products are defined by 9 CFR 431 as “canned products in which any component has a pH value above 4.6”. Since the product under study has a pH greater than 4.6, it falls within this definition. For these products, FSIS specifies thermal processing standards targeting *Clostridium botulinum* and *Clostridium sporogenes*. These standards require a 12-log reduction in *C. botulinum* spores to ensure public health protection and a 5-log reduction in *C. sporogenes* spores to achieve commercial sterility. The focus on *C. sporogenes* derives from its greater heat resistance compared to *C. botulinum*. This characteristic makes *C. sporogenes* a reliable indicator to determine whether a thermal process is sufficiently rigorous to destroy spores of other microorganisms, including *C. botulinum* and those responsible for product spoilage [68]. At the EU level, by contrast, all FBs, with the exception of primary production, are required to develop HACCP plans by conducting a hazard analysis to identify hazards that must be eliminated or reduced to acceptable levels, based on scientific evidence or the specific experience of the FBs themselves [16]. Furthermore, unlike USA legislation, which allows FBs to choose whether to include microbiological hazards in the HACCP plan for TPCS products, EU legislation does not provide such an option. In fact, according to Article 5 of Regulation (EC) No 852/2004, FBs are required to identify, within their HACCP plan, any hazard, including microbiological hazards, that is reasonably likely to occur, and to assess its relevance within the specific context of the production process. Furthermore, EU legislation, specifically Regulation (EC) No 2073/2005 and subsequent amendments and supplements on microbiological criteria for foodstuffs [69], does not establish safety or hygiene criteria for *Clostridium* spp. This specific microbiological hazard has instead been considered in an EFSA scientific opinion [67]. The opinion emphasizes the need for sufficient thermal processing to achieve an F_0_ value that ensures a 12-log reduction in *C. botulinum* as required under USA legislation. This “12-log concept” therefore represents a methodological approach shared by both the USA and EU systems, aimed at achieving a relevant reduction in *C. botulinum* spores, from one billion to one spore per thousand units, thereby safeguarding the microbiological safety of the product. This is not surprising given that both systems are based on solid historical scientific evidence and harmonized international principles [70,71]. As regards *C. sporogenes*, although the EFSA opinion does not explicitly identify this bacterium as a spoilage target, it acknowledges the importance of eliminating anaerobic spore-forming bacteria to prevent spoilage, thus aligning with the FSIS guidelines [67,72]. Therefore, as the FB under study was already applying a thermal treatment capable of ensuring microbiological safety against *Clostridium* spp., in compliance with EU legislation and, consequently, with USA microbiological safety standards, no modifications to the applied thermal treatment were required.

A key step of the HACCP system is the identification of CCPs (Principle 2) within the production process [60,73]. These are points or phases where control measures can be applied to prevent, eliminate, or reduce hazards to acceptable levels, playing a crucial role in maintaining the overall safety of the food product [59,74]. In this regard, differences were identified between the EU and USA approaches to determining CCPs. While the decision tree proposed in Commission Notice 2022/C 355/01 traces the scheme set by Codex Alimentarius [14], the USA guideline (FSIS-GD-2020-0008) presents a decision-making scheme that does not fully overlap with it. Nevertheless, both schemes lead to equivalent results and, therefore, no changes to the CCPs identified by the FB under study were required. Regarding the establishment of critical limits (Principle 3), monitoring procedures (Principle 4), and corrective actions (Principle 5), no differences were observed between the USA and EU systems that would require substantial modifications to the FB’s HACCP plan. Accordingly, these principles were not examined in detail in the present study.

#### 3.5.2. Areas Requiring Implementation: Verification Procedures (Principle 6) and Recordkeeping (Principle 7)

More substantial differences emerged with respect to verification procedures (Principle 6), particularly concerning initial validation and ongoing verifications (9 CFR 417.4 (a) (2)). Validation of a control measure is defined as the process of “obtaining evidence that a control measure or combination of control measures, if properly implemented, is capable of controlling the hazard to achieve a specified outcome” [75]. In the USA, validation is essential for the establishment of the so-called “process schedule”, a written document describing “the thermal process and any specified critical factors for a given canned product required to achieve shelf stability” (9 CFR 431.1). Critical factors are defined as “any characteristic, condition, or aspect of a product, container, or procedure that affects the adequacy of the process schedule” (9 CFR 431.1). In the case of TPCS products, such as the one under study, the relevant critical factors to be considered for the proper application of the thermal process are explicitly listed in 9 CFR 431.4. Specifically, this provision states that critical factors must be measured, controlled, and recorded by the FB at a defined frequency to ensure compliance with the limits established in the process schedule. Moreover, 9 CFR 431 specifies that the process schedule must be developed by a “processing authority” defined as “a person or organization with expert knowledge of thermal processing requirements for foods in hermetically sealed containers” (9 CFR 431.1) and that it constitutes a primary supporting document for the HACCP plan. Consequently, consistency between the process schedule and the HACCP plan is essential [76,77]. In the EU, general food hygiene legislation (Regulation (EC) No 852/2004 and further amendments and supplements) does not provide detailed guidance on the validation of thermal treatments comparable to that set out under 9 CFR regulations. However, the validation of control measures is recognized as an essential component of HACCP-based procedures, as clarified by the European Commission in its notice (Commission Notice 2022/C 355/01), which highlights that control measures must be validated and “should be supported by detailed procedures and specifications to ensure their effective implementation”. Unlike the USA regulatory framework, however, EU legislation does not formally require the development of a “process schedule” by a “processing authority”, nor does it provide a legally defined list of critical factors to be monitored, such as that established in 9 CFR 431.4. Accordingly, the FB was requested to validate its thermal process in order to demonstrate its effectiveness in controlling identified hazards, considering the “critical factors” listed in 9 CFR 431.4. This difference highlights the contrast between the USA’s prescriptive regulatory approach, characterized by standardized procedures and clearly defined responsibilities, and the EU’s outcome-based model, which affords greater autonomy and flexibility to FBs.

As mentioned above, another difference concerns ongoing verifications. Verification is defined as “the application of methods, procedures, tests, and other evaluations, in addition to monitoring, to determine whether a control measure is or has been operating as intended” [14]. Ongoing verification activities include, but are not limited to, the calibration of process-monitoring instruments, direct observations of monitoring activities and corrective actions, and the review of records generated and maintained in accordance with 417.5(a)(3). By contrast, monitoring refers to “the act of conducting a planned sequence of observations or measurements of control parameters, to assess whether a control measure is under control” [14]. Thus, while monitoring provides a real-time assessment, ongoing verification ensures that the HACCP system is working effectively on a daily basis [59]. Furthermore, the frequency of ongoing verification activities must be determined by the FB on the basis of its own risk assessment [64]. However, while USA legislation provides detailed instructions on how FBs must conduct ongoing verification activities, EU legislation (article 5 of Regulation (EC) No 852/2004) does not detail specific methods. It just requires FBs to “…establish procedures, which shall be carried out regularly, to verify that the implemented measures are working effectively”. More specific details have been subsequently provided by the Commission Notice 2022/C 355/01 [16]. Nevertheless, as a notice, this document is not legally binding, and its full implementation is therefore not mandatory, unlike the requirements laid down in 9 CFR 417. In the case of the FB under study, direct observation of monitoring activities was not included in the HACCP plan and thus represents an element requiring implementation in accordance with 9 CFR 417.4(a)(2)(ii). Specifically, these observations must be carried out by members of the HACCP team or other internal personnel, such as supervisory staff. The FB was also informed by the CA about the possibility of involving external resources or personnel not directly involved in the development or daily management of the HACCP systemin order to ensure an adequate level of independence, objectivity, and impartiality during ongoing verification activities [59,78].

**Figure 5:**
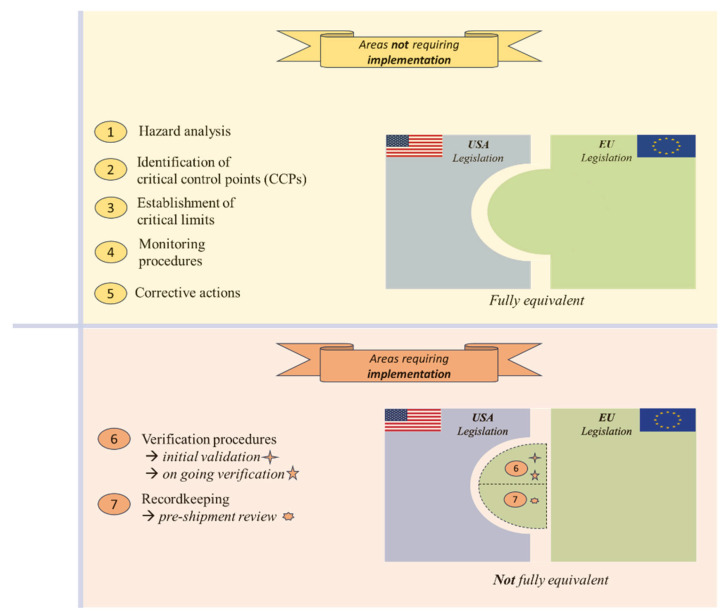
Graphical representation of the areas requiring and not requiring implementation within the Hazard Analysis and Critical Control Points (HACCP) plan. USA, United States of America; EU, European Union. Rectangles represent the USA and the EU legislation, while the overlapping area indicates equivalence between the two systems. The dotted area (**below**) highlights requirements needing implementation. Numbers correspond to the HACCP principles listed on the left.

In the USA, according to 9 CFR 417.5(c), FBs shall implement final verification/recordkeeping (Principle 7), commonly referred to as pre-shipment review. The pre-shipment review consists of a systematic examination of all the records related to each production batch prior to dispatch [79]. Its purpose is to ensure that all HACCP system requirements have been met and that the product is free from food safety hazards and other causes of adulteration, thereby making it suitable for commerce [64]. During this review, the individual designated by the FB must check the documents associated with the production of the batch intended for shipment, verifying that “all critical limits have been met and, if appropriate, corrective actions were taken, including the proper disposition of product” (9 CFR 417.5(c)). According to 9 CFR 417, the review must be dated and signed by someone other than the one who prepared the documents, preferably an individual trained according to 9 CFR 417.7 or by the responsible establishment official. In the EU, although FBs are responsible for product safety and compliance through the implementation of HACCP systems that include control measures and procedures for monitoring, verification, and recordkeeping, a formally documented and mandatory final review prior to marketing is not required. For this reason, the FB under study was requested to implement the pre-shipment review as part of the verification and recordkeeping procedures included in its HACCP plan. A graphical representation of the areas requiring and not requiring implementation within the HACCP plan is reported in Figure 5. A summary of the requirements (PRPs, SSOPs, HACCP, and thermal processing) that are shared or not considered equivalent between the EU and the USA is presented in Table 3.

### 3.6. Export Authorization Process: Procedural and Operational Barriers and Support Strategies for Small and Medium-Sized Italian FBs

**Figure 6:**
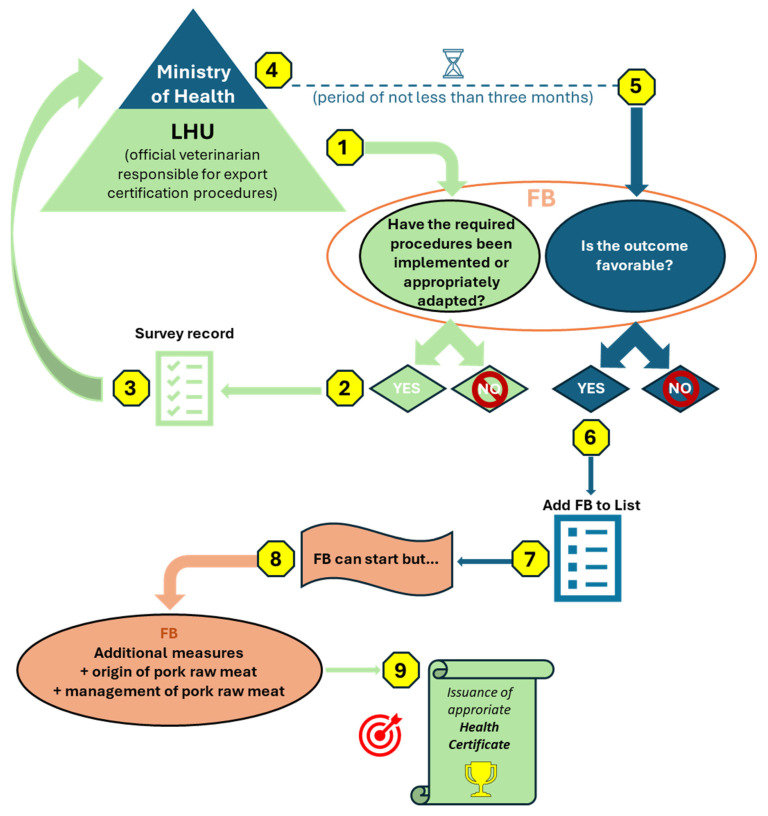
Schematic representation of the entire export authorization process. The numbered symbols (1–9) indicate the sequential steps of the procedure. Color coding identifies institutional responsibilities: green elements refer to actions performed by the Local Health Unit, blue elements refer to actions performed by the Ministry of Health, and orange elements refer to actions undertaken by the food business. LHU, Local Health Unit; Fb, food business.

By integrating regulatory analysis with FSMSs and operational practices, this study addresses a critical gap between regulatory requirements and their practical application. This approach supports a more effective translation of regulatory equivalence into establishment-level food safety management. Overall, the export authorization process for pork-based products to the USA follows a complex and multi-step pathway. In fact, once all the required procedures to align with USA legislation described in Section 3.4 and Section 3.5 have been addressed, the FB under study must submit a set of documents to the Italian Ministry of Health, through the competent LHU. These documents include the survey record carried out by the LHU itself, based on an on-site verification, attesting compliance with all the aforementioned USA requirements [46]. After submission, the FB must operate for a period of not less than three months following the attested requirements. At the end of this period, the FB is required to validate its FSMS certifying its effective implementation and effectiveness. Following this, the Italian Ministry of Health, through specially designated inspectors, conducts an additional on-site verification at the FB to verify the effective implementation of these requirements [46]. If the outcome is favorable, the FB is added to the list of establishments authorized to export to the USA, managed by the Ministry of Health itself [80]. However, inclusion in the list is not sufficient to initiate exports. The FB must also comply with specific additional requirements related to the origin and management of pork meat, such as the obligation to source it from establishments that are likewise authorized to export to the USA, and to store and handle it in clearly identified, designated areas that are physically and visually separated, to prevent any commingling with meat or other materials that do not meet USA requirements [81]. The final step consists of obtaining the appropriate health certificate (e.g., US-C01 + Annex E, US-C02, or US-C03). For pork-based pasta sauces, the appropriate health certificate model (US C01 + annex E, US-C02, or US-C03) depends on the origin of the pork raw material and on the sanitary status of the geographic areas where the FBs involved in the production process are located [82], recognized by APHIS as free from Swine Vesicular Disease (9 CFR 94.12 [83]). Figure 6 schematically illustrates the sequential process, from the verification of requirements, through inclusion in the authorized establishments list, to the issuance of the export health certificate.

While this multi-step process ensures a high level of regulatory oversight and strict alignment with USA requirements, it also adds to an already demanding preliminary phase. From an economic perspective, the export authorization process does not involve a single fixed cost, but rather a combination of direct and indirect costs that may vary considerably depending on the size and structure of the FB, its initial level of compliance, as well as the type of product to be exported and the destination country. Based on the case study and on the operational steps described in Section 3.4 and Section 3.5, the main cost components for this FB include internal organizational adjustments and staff training, the implementation, validation, and documentation of the FSMS (SSOPs and HACCP-based procedures), as well as the preparation of documentation required by the CAs.

For SMEs, these costs are generally compounded by expenses related to internal human resources involved, eventual consultancy support, and the time required to achieve and demonstrate compliance [84]. Overall, these costs are difficult to quantify. Although the administrative fees applied by the Italian CA for inclusion in the list of establishments authorized to export to the USA are fixed and relatively limited, the overall economic burden may nonetheless be relevant, particularly during the initial alignment phase. Taken together, these economic and organizational constraints may constitute a relevant barrier to market access, with the potential to delay or discourage the entry of new businesses, especially SMEs, which often have more limited organizational and technical resources than larger companies [85,86].

In addition to this complexity, potential interpretative difficulties related to ministerial circulars may also arise. As previously mentioned, these documents are internal provisions primarily addressed to the CAs [27]. Therefore, although FBs could rely on them as guidelines for implementing USA requirements, they are not originally designed for their direct use. The analysis of ministerial circulars has indeed revealed that, while they provide a general overview of the responsibilities of the main USA public authorities (FSIS, APHIS, and FDA) and of HACCP-based procedures as defined by the Codex Alimentarius [14], they offer more limited guidance on more specific operational aspects, such as SSOPs. In line with this, the study by Antoci et al. [8] identified several non-compliances in the implementation of SSOPs by certain Italian exporting FBs, most notably the absence of a documented list of food-contact surfaces to be monitored and verified. In light of this evidence, the complexity of the process and the potential difficulties in interpreting the currently available documentation may represent an additional barrier to market access. For these reasons, it would be advisable to provide structured technical and training support tools aimed at FBs, particularly SMEs, in order to reduce the risk that these critical issues could lead to exclusion from international trade. Regarding the organization of official controls and any non-compliances detected after export authorization has been granted, their organization and management are carried out within the framework of control procedures established by the CAs, in accordance with the national regulatory framework and with the operational arrangements agreed with USA authorities. In this context, the CA is required to verify the continued maintenance over time of the requirements underlying the export authorization. In cases of serious or persistent non-compliance, the CA may order the suspension or revocation of the authorization to export to the USA, including the removal of the establishment from the official list of authorized exporters. USA authorities may also intervene if non-compliances are identified during border controls or during audit activities conducted at authorized establishments, adopting measures such as the rejection of consignments or the temporary suspension of exports from the establishment or, in more serious cases, from the country concerned.

The validity, suspension, or revocation of export authorizations is therefore strictly linked to the continuous maintenance of compliance with the applicable requirements. Health certificates are issued on a consignment-by-consignment basis and are valid only when all regulatory conditions are met at the time of export, whereas establishment-level authorization remains subject to ongoing official controls. These mechanisms define a dynamic surveillance system in which market access depends on the sustained maintenance of compliance over time.

In this scenario, the crucial role played by the CA, at both central and local levels, in supporting and verifying FBs in fulfilling export requirements becomes evident. At the central level, the CA is responsible for issuing ministerial circulars and for defining the technical and regulatory framework necessary to ensure alignment with USA requirements. At the local level, the official veterinarian plays a key role in verifying the effective implementation of such requirements within FBs. Their tasks include carrying out on-site inspections, assessing compliance with the FSMS, and other specific provisions, up to the issuance of the health certificate required for export. This dual responsibility demands not only regulatory expertise but also the ability to apply complex provisions within an operational context. It is therefore essential that official veterinarians receive targeted and continuously updated training on export-related issues. Furthermore, their direct knowledge of the FB they oversee allows them to act not only as inspectors but also as technical interlocutors, addressing any interpretative gaps and guiding FBs in meeting third-country requirements, such as those of the USA. Strengthening their technical and regulatory competencies, particularly in the field of export certification, thus represents a key policy lever for improving the overall efficiency and credibility of the export process. Their dual role as controllers and facilitators indeed makes them pivotal actors in the implementation of transnational food safety requirements.

### 3.7. Regulatory Divergences and Operational Implications for Export Compliance

The case study highlighted a divergence in regulatory orientation between the EU and the USA that can also be interpreted in light of different “regulatory cultures” and institutional ways of constructing and demonstrating safety (i.e., different expectations regarding what evidence is needed and how it should be generated, documented, and verified). Within this framework, the USA approach to food safety tends to be more prescriptive and process-oriented, emphasizing formalized procedures and the use of predefined models and templates that provide a standardized pathway for demonstrating compliance. The EU framework, by contrast, is more outcome-based, focusing on the achievement of overarching hygiene and safety objectives and allowing greater flexibility in the choice of operational means, provided they are effective and justified within the specific context. This distinction cannot be attributed solely to technical differences but may also reflect cultural and historical differences in risk governance [87]. Explicitly acknowledging this difference helps interpret the gaps identified in this study and clarifies why compliance challenges arise not only from technical requirements but also from different expectations regarding how food safety should be demonstrated and verified in practice. From a broader perspective, the operational barriers observed at the establishment level also reflect the fragmentation and layering of requirements across supply chains and destination markets. In the food sector, this complexity is further intensified by product heterogeneity and the intrinsic interaction between process control and final product characteristics. As a result, even where food safety systems are well established, export compliance may require substantial effort to identify and consolidate applicable sources, formalize documentation, and adapt operational procedures, an impact that is particularly significant for SMEs. Within this context, voluntary certification schemes and standards can play a supportive role by promoting internal FSMS standardization, strengthening documentation structure, and providing a common language across the supply chain and commercial relationships. However, these tools do not replace legal requirements or official export authorization procedures, and, above all, they are not sufficient on their own to ensure compliance with all applicable requirements. Therefore, while voluntary certifications may reduce certain informational asymmetries and facilitate internal organization, they remain complementary to the obligations imposed by CAs and to country-specific conditions. This landscape is further shaped by the international dimension of regulated trade. Instruments such as the SPS Agreement within the WTO framework provide the general context in which sanitary requirements related to trade are situated. However, the present work did not aim to assess their effectiveness; rather, it highlighted that, in practice, market access is defined by specific requirements and verification activities applied at the product and establishment level. From this perspective, reducing operational uncertainty depends primarily on the availability of clear implementation criteria, accessible documentation, and technical tools that support the translation of requirements into implementable procedures, including through their consolidation and systematization into a coherent and up-to-date set of guidelines, particularly to SMEs.

### 3.8. Applicability of the Framework to Other Products and Markets

Although the analytical framework developed in this study was applied to a TPCS meat-based product intended for the USA market, its three-step structure was designed to be transferable and adaptable to other food categories and international markets. Its strength lies in the structured approach used to identify, compare, and operationalize regulatory requirements, rather than in the direct transferability of specific technical measures. When applied to other food categories or to different third-country markets, the framework requires adaptation to product-specific hazards and destination-specific regulatory conditions, while preserving the same logical sequence of analysis. Several challenges may arise in this process, including: (i) the need to address different microbiological, chemical, and processing requirements, which may vary in stringency between countries; (ii) differences in regulatory frameworks, certification procedures, and lists of authorized establishments; and (iii) different jurisdiction among public authorities responsible for food safety and hygiene, depending on the product, even within the same country (e.g., FDA rather than FSIS in the USA; see Section 3.1.1), which may affect verification procedures and required documentation. In these cases, a preliminary phase of mapping the specific requirements of the target market is essential, followed by adaptation of the concordance tables and operational procedures to the characteristics of the products and local regulations. Future applications could also benefit from more advanced evidence-gathering methods to support this mapping phase, such as automated crawling of regulatory agency websites or the use of generative Artificial Intelligence tools to systematically retrieve and classify regulatory examples and guidance. While potentially improving coverage and reducing selection bias compared to a search engine/keyword-based approach, these methods would require careful attention to source validation. This suggests that, while the general structure can be maintained, practical implementation may require relevant operational customization, highlighting the importance of methodological flexibility and adequate technical and regulatory expertise for SMEs aiming to export to different markets. In this sense, the developed framework can be considered product-independent but process-specific.

## 4. Conclusions

The analysis of a real case involving a medium-sized Italian FB, conducted through a structured and transferable analytical framework, highlighted the need to comply with specific USA regulatory requirements that go beyond those mandated by EU legislation. In particular, four key areas required implementation: (i) application of SSOPs; (ii) validation of the thermal process, including all critical factors; (iii) direct observation of monitoring and corrective actions as part of ongoing verification; (iv) implementation of the pre-shipment review. Beyond technical compliance, the export authorization process includes additional operational requirements and a mandatory preliminary operational period. These steps involve a relevant organizational and economic burden, which may represent a barrier to market access, particularly for SMEs. Based on this, key policy implications emerge: operational support for SMEs, including practical tools, and clearer implementation guidance aligned with USA standards; on the other hand, specialized and ongoing training for official veterinarians to strengthen their dual role as inspectors and technical facilitators throughout the export process. This case demonstrates that overcoming export barriers is not solely a matter of food safety, but also of building a support system that helps businesses translate complex requirements into practical, sustainable, and effective operational measures.

## Figures and Tables

**Table 1:** The United States of America and the European Union’s mandatory legislation, guidelines, directives, and other supplementary documents have been collected. The regulatory requirements reported in the table reflect the legal and administrative framework in force at the time of data collection (March–May 2023).

United States of America (USA)	
Mandatory legislation	9 CFR part 301 “Terminology; adulteration and misbranding standards”. 9 CFR part 314 “Handling and disposal of condemned or other inedible products at official establishments”. 9 CFR part 416 “Sanitation”. 9 CFR part 417 “Hazard Analysis and Critical Control Point systems”. 9 CFR part 431 “Thermally processed, commercially sterile products”.
Guidelines	FSIS-GD-1999-0003 “Update—416.2(g): water supply and water, ice, and solution reuse”. FSIS-GD-2016-0003 “Sanitation performance standards compliance guide”. FSIS-GD-2018-0005 “Meat and poultry hazard and control guide”. FSIS-GD-2020-0008 “Guidebook for the preparation of HACCP plans”. FSIS-GD-2020-0009 “A Sanitation Standard Operating Procedure model”. FSIS-GD-2021-0010 “HACCP model for thermally processed, commercially sterile product”.
Directives	FSIS Directive 5000.1 (REV8) “Verifying an establishment’s food safety system”. FSIS Directive 5000.4 (REV 3) “Performing the pre-operational Sanitation Standard Operating Procedures verification task”. FSIS Directive 7530.1 (Rev 4) “Handling a process deviation or abnormal container of thermally processed, commercially sterile canned product”. FSIS Directive 7530.2 (REV 1) “Verification activities in canning operations that choose to follow the canning regulations”.
Supplementary documents	Microbiology of thermally processed commercially sterile and shelf-stable meat and poultry products (2005). Sanitation Standard Operating Procedures (2019). Thermally processed products FSA tool VS3 (2020). Thermal processing commercially sterile self-paced training course (2021).
**European Union (EU)**	
Mandatory legislation	Regulation (EC) No 178/2002 of the European Parliament and of the Council of 28 January 2002 laying down the general principles and requirements of food law, establishing the European Food Safety Authority and laying down procedures in matters of food safety. Regulation (EC) No 852/2004 of the European Parliament and of the Council of 29 April 2004 on the hygiene of foodstuffs. Regulation (EC) No 853/2004 of the European Parliament and of the Council of 29 April 2004 laying down specific hygiene rules for food of animal origin. Regulation (EC) No 2073/2005 of 15 November 2005 on microbiological criteria for foodstuffs. Regulation (EC) No 1069/2009 of the European Parliament and of the Council of 21 October 2009 laying down health rules as regards animal by-products and derived products not intended for human consumption and repealing Regulation (EC) No 1774/2002 (Animal by-products Regulation). Regulation (EU) 2017/625 of the European Parliament and of the Council of 15 March 2017 on official controls and other official activities performed to ensure the application of food and feed law, rules on animal health and welfare, plant health, and plant protection products.
Supplementary documents	2022/C 355/01 Commission Notice on the implementation of food safety management systems covering Good Hygiene Practices and procedures based on the HACCP principles, including the facilitation/flexibility of the implementation in certain food businesses. EFSA Opinion of the Scientific Panel on Biological Hazards on the request from the Commission related to *Clostridium* spp. in foodstuffs (2005).

**Table 2:** Italian Ministry of Health circulars collected and analyzed. These documents reflect the administrative framework in force at the time of data collection (March–May 2023).

Ministerial Circulars	
Documents	Description
DGISAN 0015006-P-14/04/2016 Export to the United States of America of food of animal origin and food containing products of animal and plant origin (composite products).	The ministerial circular clarifies the responsibilities of the US public authorities (USDA and FDA) for ensuring food safety and hygiene.
DGISAN 0015012-P-14/04/2016 Procedure for registration in the USDA-FSIS list of establishments authorized to export to the United States of America.	It offers detailed instructions on the procedures to follow for inclusion in the list of establishments authorized to export food products to the United States.
DGISAN 0010140-P-17/03/2017 Official control at establishments on the list of Italian facilities authorized for the export of food products under USDA-FSIS jurisdiction in the USA—REV 1.	It is specific guidance on the methods and responsibilities of official controls at establishments authorized to export to the United States. The objective is to verify the compliance of facilities and products with the requirements established by bilateral agreements between Italy and the USA, ensuring the maintenance of equivalence between US and EU systems.
DGISAN 0040602-24/10/2018 Export to the USA of “thermally processed-commercially sterile” products.	It provides guidance on the official control of products classified as “thermally processed commercially sterile” that are exported to the USA. In addition, it offers useful guidance to Food Business Operators (FBOs) on managing HACCP plans and implementing corrective actions required in case of nonconformities affecting the product, the heat treatment process, or the container.
DGISAN 0010382-24/03/2020 Start of exports to the United States of America of composite products falling within the scope of the USDA and classified as Not Ready to Eat (NRTE) (composite products containing pork-based ingredients).	The circular establishes that the FB must ensure that the raw materials used for products intended for export to the United States come from authorized sources and are accompanied by the necessary health documentation certifying their suitability for export.
DGISAN 0015423-12/04/2022 Clarification on the use of USDA-health certificates for exports to the US of pork and pork products.	The circular provides clarification on the use of USDA health certificates required for exporting pork and pork products to the United States (US C01 + annex E, US-C02, or US-C03).

**Table 3:** Comparative summary of shared and non-equivalent food safety requirements (Prerequisite Programs (PRPs), Sanitation Standard Operating Procedures (SSOPs), Hazard Analysis and Critical Control Points (HACCP), and thermal processing) between the European Union (EU) and the United States of America (USA). CCPs, critical control points; TPCS, thermally processed and commercially sterile.

Requirement	Shared (EU and USA)	EU Only	USA Only
PRPs (animal byproducts management)	Safe handling and disposal	Risk-based categorization (Categories 1/2/3)	“Condemned” vs. “other inedible products” classification
Cleaning and sanitation procedures/SSOPs	Good hygiene practices, cleaning and sanitation	Flexibility in verification methods, outcome-based control	Specific pre-operational and operational SSOPs
HACCP implementation	Hazard analysis, CCPs, critical limits, monitoring, corrective actions	Product-specific HACCP plan development without predefined templates	Use of predefined HACCP models for product categories (e.g., TPCS), formal verification & recordkeeping, pre-shipment review
Thermal processing	Thermal treatment for microbiological safety	No specific “process schedule” requirement	Process schedule by processing authority, critical factor monitoring & validation

## Data Availability

The original contributions presented in this study are included in the article. Further inquiries can be directed to the corresponding authors.

## Supplementary Materials

The following supporting information can be downloaded at: https://www.mdpi.com/article/10.3390/foods15040761/s1. Table S1: Comparative analysis through concordance tables between USA and EU systems; Table S2: Product categorization according to 9 CFR 417(b)(1).

## Abbreviations

The following abbreviations are used in this manuscript:

APHIS	Animal and Plant Health Inspection Service
CA	Competent Authority
CCPs	Critical control points
CFR	Code of Federal Regulations
DHHS	Department of Health and Human Services
EC	European Commission
EU	European Union
FAO	Food and Agriculture Organization of the United Nations
FBs	Food businesses
FDA	Food and Drug Administration
FSIS	Food Safety and Inspection Service
FSMS	Food Safety Management System
HACCP	Hazard Analysis and Critical Control Points
IPP	Inspection Program Personnel
ISO	International Organization for Standardization
LHU	Local Health Unit
PRPs	Prerequisite Programs
SMEs	Small and medium-sized enterprises
SPS	Sanitary and Phytosanitary
SSOPs	Sanitation Standard Operating Procedures
TPCS	Thermally processed and commercially sterile
USA	United States of America
USDA	United States Department of Agriculture
WHO	World Health Organization
WOAH	World Organization for Animal Health
WTO	World Trade Organization

## References

[1] Carbone A., Henke R. (2023). Recent trends in agri-food Made in Italy exports. Agric. Food Econ..

[2] Malorgio G., Grazia C., Camanzi L. (2016). International trade regulation and food safety: The case of Italian imports of fruit and vegetables from Southern Mediterranean countries. Riv. Econ. Agrar..

[3] Othmani A., el Weriemmi M., Bakari S. (2024). Effect of food exports on economic growth: Fresh insights from Italy. J. Dev. Econ..

[4] Remondino M., Zanin A. (2022). Logistics and agri-food: Digitization to increase competitive advantage and sustainability. Literature review and the case of Italy. Sustainability.

[5] (2022). Consiglio per la Ricerca in Agricoltura e l’Analisi dell’Economia Agraria, Commercio con l’Estero dei Prodotti Agroalimentari. https://www.crea.gov.it/web/politiche-e-bioeconomia/-/rapporto-commercio-estero-prodotti-agroalimentari.

[6] (2024). Consiglio per la Ricerca in Agricoltura e l’Analisi dell’Economia Agraria, Comunicato Crea-Agritrend, Agroalimentare, II Trimestre. https://www.crea.gov.it/-/agroalimentare-ii-trimestre-2024-buon-andamento-dell-export-8-2%25-.

[7] Istituto di Servizi per il Mercato Agricolo Alimentare (2024). Report—Scambi con l’Estero. La Bilancia Agroalimentare Italiana nel I Semestre. https://www.ismeamercati.it/flex/cm/pages/ServeBLOB.php/L/IT/IDPagina/13265.

[8] Antoci S., Iannetti L., Centorotola G., Acciari V.A., Pomilio F., Daminelli P., Romanelli C., Ciorba A.B., Santini N., Torresi M. (2021). Monitoring Italian establishments exporting food of animal origin to third countries: SSOP compliance and Listeria monocytogenes and *Salmonella* spp. contamination. Food Control.

[9] Herzfeld T., Drescher L.S., Grebitus C. (2011). Cross-national adoption of private food quality standards. Food Policy.

[10] Neeliah S.A., Neeliah H., Goburdhun D. (2013). Assessing the relevance of EU SPS measures to the food export sector: Evidence from a developing agro-food exporting country. Food Policy.

[11] Neri D., Antoci S., Iannetti L., Ciorba A.B., D’Aurelio R., del Matto I., di Leonardo M., Giovannini A., Prencipe V.A., Pomilio F. (2019). EU and US control measures on *Listeria monocytogenes* and *Salmonella* spp. in certain ready-to-eat meat products: An equivalence study. Food Control.

[12] (2004). Regulation of the European Parliament and of the Council of 29 April 2004 Laying Down Specific Hygiene Rules for on the Hygiene of Foodstuffs.

[13] (2004). Regulation of the European Parliament and of the Council of 29 April 2004 on the Hygiene of Foodstuffs.

[14] General Principles of Food Hygiene. https://www.fao.org/fao-who-codexalimentarius/sh-proxy/en/?lnk=1&url=https%253A%252F%252Fworkspace.fao.org%252Fsites%252Fcodex%252FStandards%252FCXC%2B1-1969%252FCXC_001e.pdf.

[15] (2002). Regulation of the European Parliament and of the Council of 29 January 2002 Laying Down the General Principles and Requirements of Food Law, Establishing the European Food Safety Authority and Laying Down Procedures in Matters of Food Safety.

[16] (2022). Commission Notice on the Implementation of Food Safety Management Systems Covering Good Hygiene Practices and Procedures Based on the HACCP Principles, Including the Facilitation/Flexibility of the Implementation in Certain Food Businesses.

[17] (2017). Regulation (EU) 2017/625 of the European Parliament and of the Council of 15 March 2017 on Official Controls and Other Official Activities Performed to Ensure the Application of Food and Feed Law, Rules on Animal Health and Welfare, Plant Health and Plant Protection Products.

[18] Linee Guida Operative per l’Attività di Certificazione per l’Esportazione di Animali e Prodotti da Parte delle Autorità Competenti. https://www.salute.gov.it/portale/temi/p2_6.jsp?lingua=italiano&id=1156&area=sicurezzaAlimentare&menu=esportazione.

[19] Trade Agreements. https://trade.ec.europa.eu/access-to-markets/it/content/accordi-commerciali.

[20] Engler A., Nahuelhual L., Cofré G., Barrena J. (2012). How far from harmonization are sanitary, phytosanitary and quality-related standards? An exporter’s perception approach. Food Policy.

[21] Principles for Food Import and Export Inspection and Certification. https://www.fao.org/fao-who-codexalimentarius/sh-proxy/pt/?lnk=1&url=https%253A%252F%252Fworkspace.fao.org%252Fsites%252Fcodex%252FStandards%252FCXG%2B20-1995%252FCXG_020e.pdf.

[22] World Organization for Animal Health Terrestrial Animal Health Code. https://www.woah.org/en/what-we-do/standards/codes-and-manuals/terrestrial-code-online-access/.

[23] World Trade Organization Agreement on the Application of Sanitary and Phytosanitary Measures. https://www.wto.org/english/docs_e/legal_e/sps_e.htm.

[24] Principles and Guidelines for National Food Control Systems. https://www.fao.org/fao-who-codexalimentarius/sh-proxy/pt/?lnk=1&url=https%253A%252F%252Fworkspace.fao.org%252Fsites%252Fcodex%252FStandards%252FCXG%2B82-2013%252FCXG_082e.pdf.

[25] (2021). Decreto Legislativo 2 Febbraio 2021, n. 27. Disposizioni per l’Adeguamento della Normativa Nazionale alle Disposizioni del Regolamento ai Sensi dell’Articolo 12, Lettere a), b), c), d) ed e) della Legge 4 Ottobre 2019, n. 117 (21G00034); (UE) 2017/625.

[26] Amorena A.L. (2011). Le regole per l’esportazione dei prodotti alimentari. Rass. Dirit. Legis. Med. Leg. Vet..

[27] Esportazione verso Stati Uniti. https://www.salute.gov.it/portale/temi/p2_6.jsp?id=1157&area=sicurezzaAlimentare&menu=esportazione.

[28] Osservatorio Sulla Giurisprudenza Amministrativa. https://www.ildirittoamministrativo.it/archivio/allegati/OSSERVATORIO%20AMMINISTRATIVO%20AL%2031%20OTTOBRE%202010%20A%20CURA%20DI%20MARIANNA%20CAPIZZI.pdf.

[29] Teixeira S., Sampaio P. (2013). Food safety management system implementation and certification: Survey results. Total. Qual. Manag. Bus..

[30] (2003). Commission Recommendation of 6 May 2003 Concerning the Definition of Micro, Small and Medium-Sized Enterprises.

[31] Allender H.D., Buchanan S., Abdelmajid N., Arnold I., Tankson J., Carter J.M. (2020). Procedure for microbiological baseline surveys conducted by US Department of Agriculture Food Safety and Inspection Service. Food Control.

[32] Reinhard R.G., Kalinowski R.M., Bodnaruk P.W., Eifert J.D., Boyer R.R., Duncan S.E., Bailey R.H. (2018). Incidence of *Listeria* spp. in ready-to-eat food processing plant environments regulated by the U.S. Food Safety and Inspection Service and the U.S. Food and Drug Administration. J. Food Prot..

[33] Hooker N.H., Teratanavat R.P., Salin V. (2005). Crisis management effectiveness indicators for US meat and poultry recalls. Food Policy.

[34] Food & Drug Administration Investigations Operations Manual 2024. https://www.fda.gov/inspections-compliance-enforcement-and-criminal-investigations/inspection-references/investigations-operations-manual.

[35] Bruno F. (2017). Il Diritto Alimentare nel Contesto Globale: USA e UE a Confronto.

[36] Keener K.M., Kutz M. (2019). Food Regulations. Handbook of Farm, Dairy and Food Machinery Engineering.

[37] Food Safety and Inspection Service Directive & Notices—Regulations, Directives, Notices, and Policy Decisions Enable FSIS to Carry Out its Mission of Protecting Public Health. https://www.fsis.usda.gov/policy/directives-notices.

[38] Consiglio di Stato, Sezione III, Sentenza 26 Ottobre 2016, n. 4478. https://www.eius.it/giurisprudenza/2016/415.

[39] Altalex Circolari interpretative: Sussiste Giurisdizione del Giudice Amministrativo. https://www.altalex.com/documents/news/2012/10/09/circolari-interpretative-sussiste-giurisdizione-del-giudice-amministrativo.

[40] Catelani A. (2021). Le Circolari Amministrative.

[41] Ministerial Circular DGISAN 0015006-P-14/04/2016. https://www.trovanorme.salute.gov.it/norme/renderNormsanPdf?anno=0&codLeg=54662&parte=1%20&serie=.

[42] Ministerial Circular DGISAN 0010140-P-17/03/2017. https://www.trovanorme.salute.gov.it/norme/renderNormsanPdf?anno=2017&codLeg=61644&parte=1%20&serie=null.

[43] (1996). Sanitation.

[44] (1996). Hazard Analysis And Critical Control Point (HACCP) Systems.

[45] Ministerial Circular DGISAN 0040602-24/10/2018. https://www.trovanorme.salute.gov.it/norme/renderNormsanPdf?anno=2019&codLeg=71974&parte=1%20&serie=null.

[46] Ministerial Circular DGISAN 0015012-P-14/04/2016. https://www.trovanorme.salute.gov.it/norme/renderNormsanPdf?anno=2016&codLeg=54661&parte=1%20&serie=.

[47] (2009). Regulation (EC) No 1069/2009 of the European Parliament and of the Council of 21 October 2009 Laying Down Health Rules as Regards Animal By-Products and Derived Products not Intended for Human Consumption and Repealing Regulation (EC) No 1774/2002 (Animal By-Products Regulation).

[48] (1996). Terminology; Adulteration and Misbranding Standards.

[49] (1996). Handling and Disposal of Condemned or Other Inedible Products at Official Establishments.

[50] dos Santos D.A., Nunes F.L., da Silva K.O., Lobo C.M.O., Alfieri A.A., Ribeiro-Júnior J.C. (2024). Effects of industrial slicing on the microbiological quality and safety of mozzarella cheese and ham. J. Agric. Food Res..

[51] Ho K.-L.G., Sandoval A., Demirci A., Feng H., Krishnamurthy K. (2020). Sanitation Standard Operating Procedures (SSOPs). Food Safety Engineering.

[52] Ribeiro J.C., Dias B.P., Nascimento A.L.d., Silva J.P.C., Rosa F.C., de Oliveira Lobo C.M. (2024). Effects of washing sanitation standard operating procedures on the microbiological quality and safety of cattle carcasses. Food Control.

[53] Agüeria D.A., Libonatti C., Civit D. (2021). Cleaning and disinfection programmes in food establishments: A literature review on verification procedures. J. Appl. Microbiol..

[54] Ahmed A., Al-Mahmood O. (2023). Food safety programs that should be implemented in slaughterhouses: Review. J. Appl. Vet. Sci..

[55] Schmidt R.H., Pierce P.D., Lelieveld H., Holah J., Gabrić D. (2016). The Use of Standard Operating Procedures (SOPs). Handbook of Hygiene Control in the Food Industry.

[56] Food Safety and Inspection Service Sanitation Standard Operating Procedures. https://www.fsis.usda.gov/sites/default/files/media_file/2021-02/13_SSOP_student.pdf.

[57] Linee Guida sui Criteri per la Predisposizione dei Piani di Autocontrollo per l’Identificazione e la Gestione dei Pericoli Negli Stabilimenti che Trattano Alimenti di Origine Animale, di cui al Regolamento (CE) n. 853/2004. http://archivio.statoregioni.it/DettaglioDocf641.html?IdProv=10987&tipodoc=2.

[58] (2020). Sanitation Standard Operating Procedure Model.

[59] Wallace C.A., Mortimore S.E., Lelieveld H., Holah J., Gabrić D. (2016). HACCP. Handbook of Hygiene Control in the Food Industry.

[60] Ngure F.M., Makule E., Mgongo W., Phillips E., Kassim N., Stoltzfus R., Nelson R. (2024). Processing complementary foods to reduce mycotoxins in a medium scale Tanzanian mill: A hazard analysis critical control point (HACCP) approach. Food Control.

[61] Radu E., Dima A., Dobrota E.M., Badea A.M., Madsen D.Ø., Dobrin C., Stanciu S. (2023). Global trends and research hotspots on HACCP and modern quality management systems in the food industry. Heliyon.

[62] (2018). Food safety Management Systems: Requirements for any Organization in the Food Chain.

[63] Jongwanich J. (2009). The impact of food safety standards on processed food exports from developing countries. Food Policy.

[64] (2020). Guidebook for the Preparation of HACCP Plans.

[65] (2018). Thermally Processed, Commercially Sterile Products.

[66] (2021). Food Safety and Inspection Service. HACCP Model for Thermally Processed, Commercially Sterile Product. https://www.fsis.usda.gov/sites/default/files/media_file/2021-08/FSIS-GD-2021-0010_0.pdf.

[67] Food Safety and Inspection Service Microbiology of Thermally Processed Commercially Sterile and Shelf-Stable Meat and Poultry Products. https://www.fsis.usda.gov/sites/default/files/media_file/2021-03/VTP_Reference_Material.pdf.

[68] Anderson N.M., Larkin J.W., Cole M.B., Skinner G.E., Whiting R.C., Gorris L.G.M., Rodriguez A., Buchanan R., Stewart C.M., Hanlin J.H. (2011). Food safety objective approach for controlling *Clostridium botulinum* growth and toxin production in commercially sterile foods. J. Food Prot..

[69] (2005). Commission Regulation (EC) No 2073/2005 of 15 November 2005 on Microbiological Criteria for Foodstuffs.

[70] (2011). Code of Hygienic Practice for Low and Acidified Low Acid Canned Foods.

[71] Food and Agriculture Organization of the United Nations Canning Principles. https://www.fao.org/4/R6918E/R6918E02.htm.

[72] European Food Safety Authority (EFSA) (2005). Opinion of the Scientific Panel on biological hazards (BIOHAZ) Related to *Clostridium* spp. in Foodstuffs. EFSA J..

[73] Garcia Martinez M., Fearne A., Caswell J.A., Henson S. (2007). Co-regulation as a possible model for food safety governance: Opportunities for public–private partnerships. Food Policy.

[74] Glowicz J., Benowitz I., Arduino M.J., Li R., Wu K., Jordan A., Toda M., Garner K., Gold J.A.W. (2022). Keeping health care linens clean: Underrecognized hazards and critical control points to avoid contamination of laundered health care textiles. Am. J. Infect. Control.

[75] Guidelines for the Validation of Food Safety Control Measures. https://www.fao.org/fao-who-codexalimentarius/sh-proxy/en/?lnk=1&url=https%253A%252F%252Fworkspace.fao.org%252Fsites%252Fcodex%252FStandards%252FCXG%2B69-2008%252FCXG_069e.pdf.

[76] Food Safety and Inspection Service Thermally Processed Products FSA Tool VS3. https://www.fsis.usda.gov/sites/default/files/media_file/2020-08/Thermally-Processed.pdf.

[77] Thermal Processing Commercially Sterile Self-Paced Training Course. https://fsistraining.fsis.usda.gov/pluginfile.php/18440/mod_resource/content/1/TPSP%20Student%20Guide%20Edits12092021.pdf.

[78] Manning L. (2013). Development of a food safety verification risk model. Br. Food J..

[79] Eubanks L., Carr C., Schmidt R.H. (2009). Hazard Analysis Critical Control Points (HACCP) Principle 7: Establish record keeping and documentation procedures. J. Dairy Sci..

[80] Stabilimenti Autorizzati all’Export di Prodotti di Origine Animale verso gli Stati Uniti d’America. https://www.salute.gov.it/consultazioneStabilimenti/ConsultazioneStabilimentiServlet?ACTION=gestioneSingoloPaese&naz=US.

[81] Ministerial Circular DGISAN 0010382-24/03/2020. https://www.aulss7.veneto.it/index.cfm?method=mys.apridoc&iddoc=7523.

[82] Ministerial Circular DGISAN 0015423-12/04/2022. https://www.aulss7.veneto.it/index.cfm?method=mys.apridoc&iddoc=7751.

[83] (2023). Foot-and-Mouth Disease, Newcastle Disease, Highly Pathogenic Avian Influenza, African Swine Fever, Classical Swine Fever, Swine Vesicular Disease, and Bovine Spongiform Encephalopathy: Prohibited and Restricted Importations.

[84] Lee J.C., Neonaki M., Alexopoulos A., Varzakas T. (2023). Case studies of small-medium food enterprises around the world: Major constraints and benefits from the implementation of food safety management systems. Foods.

[85] Organization for Economic Co-Operation and Development (2023). OECD SME and Entrepreneurship Outlook.

[86] Paul J., Parthasarathy S., Gupta P. (2017). Exporting challenges of SMEs: A review and future research agenda. J. World Bus..

[87] Echols M.A. (1998). Food safety regulation in the European Union and the United States: Different cultures, different laws. Colum. J. Eur. L..

