# Supplementary material for: A Structured Analytical Framework to Facilitate EU Food Exports to the USA: A Case Study Analyzing Barriers and Support Strategies
Source: Foods. 2026 Feb 19;15(4):761. doi: 10.3390/foods15040761 (PMC12940801; doi:10.3390/foods15040761)
Supplement: Supplementary file 1 [file foods-15-00761-s001.zip › Supplementary Materials.pdf]

Table S1. Comparative analysis through concordance tables between USA and EU systems.

9 CFR part 416 SPS	USA GUIDELINES DIRECTIVES and other supplementary documents<br>• *FSIS-GD-1999-0003*<br>• *FSIS-GD-2016-0003*<br>• *FSIS DIRECTIVE 5000.1 (REV.8)*<br>+<br>*9 CFR part 301*<br>*9 CFR part 314*	EU LEGISLATION<br>• *Regulation (EC) 852/2004*<br>• *Regulation (EC) 853/2004*<br>• *Regulation (EC) 1069/2009*<br>• *Regulation (EU) 2017/625*<br>• *Regulation (EU) 2019/627*<br>• *Directive (EU) 2020/2184*	EU SUPPLEMENTARY DOCUMENTS<br>• *Commission Notice 2022/C 355/01*	IMPLEMENTATION?
**416.1 *GENERAL RULES***		***Reg. (EC) 852/2004***		
Each official establishment must be operated and maintained in a manner sufficient to prevent the creation of insanitary conditions and to ensure that product is not adulterated.	//	***Article 3:***<br>*Food business operators shall ensure that all stages of production, processing and distribution of food under their control satisfy the relevant hygiene requirements laid down in this Regulation.*	//	***NO***
416.2 ESTABLISHMENT GROUNDS AND FACILITIES	***FSIS DIRECTIVE 5000.1 (REV.8)***<br>***FSIS-GD-1999-0003***<br>***FSIS-GD-2016-0003***<br>+<br>***9 CFR part 301***<br>***9 CFR part 314***	***Reg. (EC) 852/2004***<br>***Reg. (EC) 853/2004***<br>***Reg. (EC) 1069/2009***<br>***Directive (EU) 2020/2184***	***Commission Notice 2022/C 355/01***	
**a) Grounds and pest control.**<br>*The grounds about an establishment must be maintained to prevent conditions that could lead to insanitary conditions, adulteration of product, or interfere with inspection*	***FSIS DIRECTIVE 5000.1 (REV.8)***<br>***III. Grounds and pest control***<br>*Although an establishment must have a pest management program, it need not be written. If establishment management decides to have a written*	***Reg (EC) 852/2004, Annex II, chapter I, paragraph 2:***<br>*The layout, design, construction, siting and size of food premises are to:*	***3.1 Infrastructure (building, equipment)***<br>*a) When assessing the risk from the location and surrounding areas, the proximity of potential sources of contamination, water supply,*	***NO***

by FSIS program employees. Establishments must have in place a pest management program to prevent the harborage and breeding of pests on the grounds and within establishment facilities. Pest control substances used must be safe and effective under the conditions of use and not be applied or stored in a manner that will result in the adulteration of product or the creation of insanitary conditions.	*program, it may or may not be included in the Sanitation SOP. If the establishment has included a written pest management program as part of the Sanitation SOP, IPP are to verify that the procedures in the Sanitation SOP are being implemented and monitored, that the establishment is documenting in the Sanitation SOP records the monitoring of the procedures, and that any necessary corrective actions are taken.*<br>*One or more of the following findings provide evidence that the establishment does not comply with 9 CFR 416.2(a):*<br>*1. There are areas around or within the establishment that allow harborage or breeding of pests. These might include tall weeds, discarded equipment, poorly maintained trash receptacles, or*<br>*similar situations close to the establishment.*<br>*2. There is evidence of pests or pest activity within the establishment (e.g., rodent droppings or flies in production areas).*<br>*3. Establishment management is unable to demonstrate that pest control substances are safe under*<br>*the conditions of use.*<br>*4. Establishment employees do not use pest control substances in accordance with label directions.*<br>*5. Pest control substances are used or stored in a manner that results in insanitary conditions.*<br>*6. There is any other condition on the grounds of the establishment that results in insanitary*<br>*conditions within the establishment.*	***a)*** *permit adequate maintenance, cleaning and/or disinfection, avoid or minimize air-borne contamination, and provide adequate working space to allow for the hygienic performance of all operations.*<br>***c)*** *permit good food hygiene practices, including protection against contamination and, in particular, pest control.*<br>***Reg. (EC) 852/2004, Annex II, chapter II, paragraph 1:***<br>*In rooms where food is prepared, treated or processed (excluding dining areas and those premises specified in Chapter III, but including rooms contained in means of transport) the design and layout are to permit good food hygiene practices, including protection against contamination between and during operations. In particular:*<br>***d)*** *windows and other openings are to be constructed to prevent the accumulation of dirt. Those which can be opened to the outside environment are, where necessary, to be fitted with insect-proof screens which can be easily removed for cleaning. Where open windows would result in contamination, windows are to remain closed and fixed during production.*<br>***Reg. (EC) 852/2004, Annex II, chapter IX, paragraph 4:***<br>*Adequate procedures are to be in place to control pests. Adequate procedures are also to be in place to prevent domestic animals from having access to places where food is prepared, handled or stored (or, where the competent authority so permits in special cases, to prevent such access from resulting in contamination).*	*wastewater removal, power supply, access for transport, climate, possible flooding, etc. should be taken into account. This should also be considered for primary production (fields).*<br>*j) Barriers should be in place to avoid access of stray animals.*<br>***3.3 Pest control: emphasis on preventive activities***<br>*a) External walls should be free of cracks or chinks, surroundings should be neat and free from debris which could provide harborage from pests, and areas for cleaning should be accessible. Access by pets or wild animals must be prohibited/prevented.*<br>*b) Insect screen should be placed at windows. When electronic devices are used for insect control, the device has to be used according to its specification.*<br>*c) Doors should be kept closed except when loading and/or unloading. Gaps between doors and floors should be pest-proofed.*<br>*d) Unused equipment and rooms should be kept clean.*<br>*e) The presence of an indoor pool of water should be addressed as soon as possible. Ponding or pooling of water must be prevented or avoided.*<br>*f) A pest control programme should be available:*<br>*i. Baits and traps (inside/outside) should be considered in appropriate numbers and also their strategic placement;*<br>*ii. the programme should cover rodents, crawling, walking and flying pests;*<br>*iii. dead pests and insects should be frequently removed*	***NO***

		Reg. (EC) 852/2004, Annex II, chapter IX, paragraph 8:<br>*Hazardous and/or inedible substances, including animal feed, are to be adequately labelled and stored in separate and secure containers.*	*ensuring no possible contact with food;*<br>*iv. the cause should be determined in case of a recurrent problem;*<br>*v. chemicals used to control harmful organisms have to be authorized by the Biocidal Products Regulation (Reg. UE n. 528/2012). Pesticides should be stored safely and used so that there is no possible contact with, inter alia, food, packaging material and equipment. Fly traps (including electric fly killers) should not be placed directly above areas where food is processed or stored;*<br>*vi. chemical substances (e.g. biocidal products used for the control rodents) should not be used to monitor the occurrence of pests but restricted to pest control activities only;*<br>*vii. professional pest control is preferable, but in most cases, it is not compulsory, provided the staff can demonstrate competence. In particular, small business can apply this flexibility.*	
**b)** ***Construction***<br>*1. Establishment buildings, including their structures, rooms, and compartments must be of sound construction, be kept in good repair, and be of sufficient size to allow for processing, handling, and storage of product in a manner that does not result in product adulteration or the*	//	***Reg. (EC) 852/2004, Annex II, chapter I, paragraph 1:***<br>*Food premises are to be kept clean and maintained in good repair and condition.*<br><br>***Reg. (EC) 852/2004, Annex II, chapter I, paragraph 2:***<br>*The layout, design, construction, siting and size of food premises are to:*	//	***NO***

creation of insanitary conditions.		***a)*** *permit adequate maintenance, cleaning and/or disinfection, avoid or minimize air-borne contamination, and provide adequate working space to allow for the hygienic performance of all operations;*<br>***b)*** *be such as to protect against the accumulation of dirt, contact with toxic materials, the shedding of particles into food and the formation of condensation or undesirable mold on surfaces;*<br>***d)*** *where necessary, provide suitable temperature-controlled handling and storage conditions of sufficient capacity for maintaining foodstuffs at appropriate temperatures and designed to allow those temperatures to be monitored and, where necessary, recorded.*<br><br>***Reg. (EC) 853/2004, section V:***<br>*Food business operators must ensure that they:*<br>*1) are constructed so as to avoid contamination of meat and products, in particular by:*<br>*(a) allowing constant progress of the operations; or*<br>*(b) ensuring separation between the different production batches.*		
2. Walls, floors, and ceilings within establishments must be built of durable materials impervious to moisture and be cleaned and sanitized as necessary to prevent adulteration of product or the creation of insanitary conditions.	**FSIS-GD-2016-0003**<br>*Floors should be made with a suitable slope to ensure drainage. The floor and wall junctures shall be covered and sealed.*	***Reg. (EC) 852/2004, Annex II, chapter II, paragraph 1, letters a, b, c:***<br>***a)*** *floor surfaces are to be maintained in a sound condition and be easy to clean and, where necessary, to disinfect. This will require the use of impervious, non-absorbent, washable and non-toxic materials unless food business operators can satisfy the competent authority that other materials used are appropriate. Where appropriate, floors are to allow adequate surface drainage;*	*//*	***NO***

		b) *wall surfaces are to be maintained in a sound condition and be easy to clean and, where necessary, to disinfect. This will require the use of impervious, non-absorbent, washable and non-toxic materials and require a smooth surface up to a height appropriate for the operations unless food business operators can satisfy the competent authority that other materials used are appropriate;*<br>***c)*** *ceilings (or, where there are no ceilings, the interior surface of the roof) and overhead fixtures are to be constructed and finished so as to prevent the accumulation of dirt and to reduce condensation, the growth of undesirable mold and the shedding of particles.*		
3. Walls, floors, ceilings, doors, windows, and other outside openings must be constructed and maintained to prevent the entrance of vermin, such as flies, rats, and mice.	*//*	***Reg. (EC) 852/2004, Annex II, chapter I, paragraph 2:***<br>*The layout, design, construction, siting and size of food premises are to:*<br>***c)*** *permit good food hygiene practices, including protection against contamination and, in particular, pest control.*<br><br>***Reg. (EC) 852/2004, Annex II, chapter II, paragraph 1, letter d:***<br>***d)*** *windows and other openings are to be constructed to prevent the accumulation of dirt. Those which can be opened to the outside environment are, where necessary, to be fitted with insect-proof screens which can be easily removed for cleaning. Where open windows would result in contamination, windows are to remain closed and fixed during production.*	*//*	***NO***

4. *Rooms or compartments in which edible product is processed, handled, or stored must be separate and distinct from rooms or compartments in which inedible product* is processed, handled, or stored, to the extent necessary to prevent product adulteration and the creation of insanitary conditions.*<br><br>****9 CFR 301.2***<br>*-Inedible product: adulterated, uninspected, or not intended for use as human food.*<br><br>*-Meat byproduct: any part capable of use as human food, other than meat, which has been derived from one or more cattle, sheep, swine, or goat.*	**FSIS-GD-2016-0003**<br>*FSIS has allowed and will continue to allow edible and inedible products to be processed, handled, or stored in the same room or area, provided that measures are taken to prevent the adulteration of the edible product or insanitation that could lead to product adulteration. Typically, if an establishment processes, handles, or stores edible and inedible products in the same room or area, the products are separated by time and/or space and stringent controls are in place to prevent product adulteration.*	***Reg. (EC) 852/2004, Annex II, chapter II, paragraph 1:***<br>*In rooms where food is prepared, treated or processed (excluding dining areas and those premises specified in Chapter III, but including rooms contained in means of transport) the design and layout are to permit good food hygiene practices, including protection against contamination between and during operations.*<br><br>***Reg. (EC) 1069/2009, Article 3:***<br>*'animal by-products' means entire bodies or parts of animals, products of animal origin or other products obtained from animals, which are not intended for human consumption, including oocytes, embryos and semen.*	***3.1 Infrastructure (building, equipment)***<br>***b)*** *Lay-out should strictly separate contaminated (high risk) from clean areas (low risk) (or there should be a separation in time and suitable cleaning in between); suitable arrangements of rooms should be made for one-direction production flow and cooled rooms or heating facilities should be insulated.*<br>***f)*** *Clearly defined storage facilities should be available for raw material, and receptacles for food and packaging materials. Only products that may be added to food (e.g. additives) should be stored in the area with the food, excluding common storage with toxic products (e.g. pesticides).*<br><br>***3.11 Personal (hygiene, health status):***<br>***e)*** *Eating, drinking and/or smoking rooms should be separated and clean.*	***NO***<br>***(but different naming and categorization of animal byproducts)***<br><br>***Note 1:*** *Reg (EC) 852/2004 does not expressly mention the separation and distinction of areas between edible and non-edible product but a separation between contaminated and clean areas is made explicit in Commission Communication 2022/C 355/01*<br>***Note 2: 9 CFR part 314 Handling and disposal of condemned or other inedible products at official establishments*** *does not classifies animal byproducts according to the same specific risk level categories established by Regulation (EC) 1069/2009 (Category 1, 2 and 3) but distinguishes them between "condemned" and "other inedible products".*
**c) Light**<br>*Lighting of good quality and sufficient intensity to ensure that sanitary conditions are maintained and that product is not adulterated must be provided in areas where food is processed, handled, stored, or examined; where equipment and utensils are cleaned; and in hand-washing areas, dressing and locker rooms, and toilets.*	***FSIS-GD-2016-0003***<br>*Recommendations are given regarding light intensity and placement of light sources in specific areas of the plant.*<br><br>• *At least 110 lux (10 foot candles) at a distance of 75 cm (30 inches) above the floor, in walk-in refrigeration units and dry food storage areas and in other areas and rooms during periods of cleaning;*<br><br>• *At least 220 lux (20 foot candles):*<br><br>*- Inside equipment such as reach-in and under-counter refrigerators;*	***Reg. (EC) 852/2004, Annex II, chapter I, paragraph 7:***<br>*Food premises are to have adequate natural and/or artificial lighting.*	***3.1 Infrastructure (building, equipment):***<br>***e)*** *There should be sufficient lighting in* ***all areas****, with special attention paid to the provision of suitable lighting to food preparation and inspection areas. Lighting should be easy to clean, with protective covers to prevent contamination of food in the event of lights breaking.*	***NO***<br><br>***Note:*** *In the European context, although no precise minimum lux is given to ensure, there is talk of ensuring "sufficient lighting," implicitly incorporating that this should ensure the adequacy of all operations, including inspection.*

	- *At a distance of 75 cm (30 inches) above the floor in areas used for handwashing, warewashing, and equipment and utensil storage, and in toilet rooms; and*<br>• *At least 540 lux (50 foot candles) at a surface where a food employee is working with food or working with utensils or equipment such as knives, slicers, grinders, or saws where employee safety is a factor.*			
**d) Ventilation**<br>*Ventilation adequate to control odors, vapors, and condensation to the extent necessary to prevent adulteration of product and the creation of insanitary conditions must be provided.*	***FSIS-GD-2016-0003***<br>*In regard to condensation, keep in mind that some forms are unavoidable and acceptable within a food processing environment, since they will neither adulterate product nor create insanitary conditions. Other forms of condensation are expected, but must be controlled by the establishment, and others are unacceptable at any time.*<br>*In some situations, condensation clearly adulterates product, creates insanitary conditions, and/or interferes with inspection.*<br>*Some examples follow:*<br>*1. Heavily beaded condensation forms on a ceiling or wall of a processing area that is not regularly cleaned and sanitized in accordance with the establishment's SSOP's (an insanitary condition is created that could lead to the adulteration of product).*<br>*2. Condensate from a cooler ceiling drips onto carcasses.*<br>*3. Condensate from refrigeration unit surfaces, which have not been cleaned and sanitized, drips onto exposed product.*	***Reg. (EC) 852/2004, Annex II, chapter I, paragraph 5:***<br>*There is to be suitable and sufficient means of natural or mechanical ventilation. Mechanical airflow from a contaminated area to a clean area is to be avoided. Ventilation systems are to be so constructed as to enable filters and other parts requiring cleaning or replacement to be readily accessible.*<br>***Reg. (EC) 852/2004, Annex II, chapter I, paragraph 6:***<br>*Sanitary conveniences are to have adequate natural or mechanical ventilation.*<br><br>***Reg. (EC) 853/2004, Annex III, Section I, chapter VII (Storage and Trasport):***<br>*Food business operators must ensure that the storage and transport of meat of domestic ungulates takes place in accordance with the following requirements*<br>*b) during the chilling operations, there must be adequate ventilation to prevent condensation on the surface of the meat.*	***3.10 Air control:***<br>***e)*** *Ventilation systems should be robust and reliable. Ventilation systems should be kept clean, so that they do not become a source of contamination. For high risk/care areas requiring air control, the implementation of positive air pressure systems and appropriate air filtering systems should be considered;*<br>***f)*** *condensation is mostly the result of poor ventilation. Condensation should be avoided in areas where food is being produced, handled or stored, especially if exposed or not packed.*	***NO***

	4. Condensate from a loading dock ceiling or wall drips onto boxes of boneless beef, breaking down the packaging.			
**e) Plumbing**<br>Plumbing systems must be installed and maintained to:<br><br>1. *carry sufficient quantities of water to required locations throughout the establishment;*	//	***Reg. (EC) 852/2004, Annex II, chapter VII, paragraph 1:***<br><br>***a)*** *There is to be an adequate supply of potable water, which is to be used whenever necessary to ensure that foodstuffs are not contaminated.*	//	***NO***
2. *properly convey sewage and liquid disposable waste from the establishment;*	//	***Reg. (EC) 852/2004, Annex II, chapter I, paragraph 8:***<br>*Drainage facilities are to be adequate for the purpose intended. They are to be designed and constructed to avoid the risk of contamination. Where drainage channels are fully or partially open, they are to be so designed as to ensure that waste does not flow from a contaminated area towards or into a clean area, in particular an area where foods likely to present a high risk to the final consumer are handled.*	//	***NO***
3. *prevent adulteration of product, water supplies, equipment, and utensils and prevent the creation of insanitary conditions throughout the establishment;*	**FSIS-GD-2016-0003**<br>*A water filter, screen, and other water conditioning device installed on water lines shall be designed to facilitate disassembly for periodic servicing and cleaning. A water filter element shall be of the replaceable type.*	***Directive (EU) 2020/2184***<br>***Article 11: Minimum hygiene requirements for materials that come into contact with water intended for human consumption***<br>*1: ….. the treatment, storage or distribution of water intended for human consumption and that come into contact with such water do not:*<br>*a) directly or indirectly compromise the protection of human health as provided for by this Directive;*<br>*b) adversely affect the color, odor or taste of the water;*<br>*c) enhance microbial growth;*<br>*d) (d) leach contaminants into the water at levels that are higher than*	//	***NO***

		necessary in view of the intended purpose of the material.		
4. *provide adequate floor drainage in all areas where floors are subject to flooding-type cleaning or where normal operations release or discharge water or other liquid waste on the floor;*	//	***Reg. (EC) 852/2004, Annex II, chapter II, paragraph 1:*** ***a)*** *Where appropriate, floors are to allow adequate surface drainage.*	//	***NO***
5. *prevent back-flow conditions in and cross-connection between piping systems that discharge waste water or sewage and piping systems that carry water for product manufacturing;* 6. *prevent the backup of sewer gases.*	//	***Reg. (EC) 852/2004, Annex II, chapter I, paragraph 8:*** ***…omissis…**. Where drainage channels are fully or partially open, they are to be so designed as to ensure that waste does not flow from a contaminated area towards or into a clean area, in particular an area where foods likely to present a high risk to the final consumer are handled.* ***Reg. (EC) 852/2004, Annex II, chapter VII, paragraph 2:*** *Where non-potable water is used, for example for fire control, steam production, refrigeration and other similar purposes, it is to circulate in a separate duly identified system. Non-potable water is not to connect with, or allow reflux into, potable water systems.*	//	***NO***
**f) Sewage disposal.** *Sewage must be disposed into a sewage system separate from all other drainage lines or disposed of through other means sufficient to prevent backup of sewage into areas where product is processed, handled, or stored. When the sewage disposal system is a private system requiring approval by a State or local health authority, the establishment must furnish FSIS with the letter of*	//	***Reg. (EC) 852/2004, Annex II, chapter I, paragraph 8:*** *Drainage facilities are to be adequate for the purpose intended. They are to be designed and constructed to avoid the risk of contamination. Where drainage channels are fully or partially open, they are to be so designed as to ensure that waste does not flow from a contaminated area towards or into a clean area, in particular an area where foods likely to present a high*	//	***NO***

approval from that authority upon request.		*risk to the final consumer are handled.*		
**g) Water supply and water, ice, and solution reuse.**<br><br>1. *A supply of running water that complies with the National Primary Drinking Water regulations (40 CFR part 141), at a suitable temperature and under pressure as needed, must be provided in all areas where required (for processing product, for cleaning rooms and equipment, utensils, and packaging materials, for employee sanitary facilities, etc.).*	//	***Reg. (EC) 852/2004, Annex II, chapter VII, paragraph 1:***<br>***a)*** *There is to be an adequate supply of potable water, which is to be used whenever necessary to ensure that foodstuffs are not contaminated.*<br>***Reg. (EC) 852/2004, Annex II, chapter I, paragraph 4:***<br>*An adequate number of washbasins is to be available, suitably located and designated for cleaning hands. Washbasins for cleaning hands are to be provided with hot and cold running water, materials for cleaning hands and for hygienic drying. Where necessary, the facilities for washing food are to be separate from the hand-washing facility.*<br>***Reg. (EC) 852/2004, Annex II, chapter II, paragraph 2:***<br>*Adequate facilities are to be provided, where necessary, for the cleaning, disinfecting and storage of working utensils and equipment. These facilities are to be constructed of corrosion-resistant materials, be easy to clean and have an adequate supply of hot and cold water.*<br>***Reg. (EC) 852/2004, Annex II, chapter II, paragraph 3:***<br>*Adequate provision is to be made, where necessary, for washing food. Every sink or other such facility provided for the washing of food is to have an adequate supply of hot and/or cold potable water consistent with the requirements of Chapter VII and be kept clean and, where necessary, disinfected.*<br><br>***Reg. (EC) 853/2004, Article 3: General obligations:***	***3.10 Water control:***<br>***a)*** *Regular own microbiological and chemical analysis of water directly in contact with food (unless community potable water) should be carried out. Factors such as the source, intended use of the water, etc. will determine the frequency of analysis;*<br>***b)*** *If community water is held in a tank prior to use, the tank must be part of a regular cleaning schedule;*<br>***c)*** *As a general rule, only potable water may be used on food of animal origin. At least clean water or where applicable clean sea water should be used in other cases;*<br>***d)*** *Control of water is an important way of controlling microbiological and chemical hazards in the primary production of fruit and vegetables (irrigation, washing at harvest)… Potable water is strongly recommended in washing of fruit and vegetables for direct consumption.*	***NO***

If an establishment uses a municipal water supply, it must make available to FSIS, upon request, a water report, issued under the authority of the State or local health agency, certifying or attesting to the potability of the water supply. If an establishment uses a private well for its water supply, it must make available to FSIS, upon request, documentation certifying the potability of the water supply that has been renewed at least semi-annually.		*Food business operators shall not use any substance other than potable water or, when Regulation (EC) 852/2004 or this Regulation permits its use, clean water, to remove surface contamination from products of animal origin, unless use of the substance has been approved by the Commission. For that purpose the Commission is empowered to adopt delegated acts in accordance with Article 11a supplementing this Regulation. Food business operators shall also comply with any conditions for use that may be adopted under the same procedure. The use of an approved substance shall not affect the food business operator's duty to comply with the requirements of this Regulation.*<br><br>***Directive (EU) 2020/2184, Article 2, Definitions:***<br>*For the purposes of this Directive, the following definitions apply:*<br>*1. 'water intended for human consumption' means:*<br>*a) all water, either in its original state or after treatment, intended for drinking, cooking, food preparation or other domestic purposes in both public and private premises, regardless of its origin and whether it is supplied from a distribution network, supplied from a tanker or put into bottles or containers, including spring waters;*<br>*b) all water used in any food business for the manufacture, processing, preservation or marketing of products or substances intended for human consumption.*<br><br>***Directive (EU) 2020/2184, Article 4 General obligations:***		

		1. Without prejudice to their obligations under other Union law, Member States shall take the measures necessary to ensure that water intended for human consumption is wholesome and clean. For the purposes of the minimum requirements of this Directive, water intended for human consumption shall be wholesome and clean if all the following requirements are met:<br>*a) that water is free from any micro-organisms and parasites and from any substances which, in numbers or concentrations, constitute a potential danger to human health;*<br>*b) that water meets the minimum requirements set out in Parts A, B and D of Annex I;*<br>*c) Member States have taken all other measures necessary to comply with Articles 5 to 14.*<br>***Directive (EU) 2020/2184, Article 6***<br>***Point of compliance***<br>*1. The parametric values set in accordance with Article 5 for the parameters listed in Parts A and B of Annex I shall be complied with:*<br>*d) in the case of water intended for human consumption used in a food business, at the point at which the water is used in that business.*		
2. Water, ice, and solutions (such as brine, liquid smoke, or propylene glycol) used to chill or cook ready-to-eat product may be reused for the same purpose, provided that they are maintained free of pathogenic organisms and fecal coliform organisms and that other physical, chemical, and microbiological contamination have been	***FSIS-GD-1999-0003***<br>*ICE REUSE*<br>*Establish a procedure to assure that ice is collected and held in a container that drains freely and in a sanitary manner. The procedure should address collection and washing of ice before it is reused.*<br>*Establish a procedure for identifying reused ice from fresh ice.*<br>*The ice or the product should be packaged in an impervious, sealed container, such as a plastic bag, to*	***Reg. (EC) 852/2004, Annex II, chapter VII,***<br>***paragraph 3.*** *Recycled water used in processing or as an ingredient is not to present a risk of contamination. It is to be of the same standard as potable water, unless the competent authority is satisfied that the quality of the water cannot affect the wholesomeness of the foodstuff in its finished form.*<br>***paragraph 4.*** *Ice which comes into contact with food or which may*	*//*	***NO***

reduced to prevent adulteration of product.<br><br>3. *Water, ice, and solutions used to chill or wash raw product may be reused for the same purpose provided that measures are taken to reduce physical, chemical, and microbiological contamination so as to prevent contamination or adulteration of product. Reuse that which has come into contact with raw product may not be used on ready-to-eat product.*<br><br>4. *Reconditioned water that has never contained human waste and that has been treated by an onsite advanced wastewater treatment facility may be used on raw product, except in product formulation, and throughout the facility in edible and inedible production areas, provided that measures are taken to ensure that this water meets the criteria prescribed in <u>paragraph (g)(1)</u> of this section. Product, facilities, equipment, and utensils coming in contact with this water must undergo a separate final rinse with non-reconditioned water that meets the criteria prescribed in <u>paragraph (g)(1)</u> of this section.*<br><br>5. *Any water that has never contained human waste and that is free of pathogenic organisms may be used in edible and inedible product*	*prevent direct contact between the product and ice.*<br>*Ice used on raw product should not be reused on any heat processed partially- or fully-cooked product.*<br>*If the ice should be free of any observable foreign material. If the ice is washed, continuous drainage should be maintained during the washing procedure. Ice from damaged containers should not be used.*<br>*Establish procedures to correct deficiencies that occur and to prevent reoccurrence.*<br><br>*<u>BRINE REUSE</u>*<br>*Brine may be reused d to chill cooked product for various lengths of time based on the type of casing, salinity, and temperature. Brine solution that is reused to chill raw or heat -treated, raw but not fully cooked product (example- e.g., smoked bacon) should be reconditioned in a manner to prevent the brine solution from becoming contaminated and adulterating the product.*<br>*Brine reuse to chill raw product should follow the same criteria as brine reused to chill heat-treated, not fully cooked product.*<br>*Establish procedures for monitoring the temperature, salinity, and free chlorine concentration of the brine being reused to chill heat-treated product. Establish an ongoing microbiological plan to ensure that the brine solution is maintained pathogen free. The monitoring plan should cover the type and frequency of any microbiological analysis, and action limits (upper/lower control limits), and actions taken to ensure product safety when those limits are exceeded.*	*contaminate food is to be made from potable water or, when used to chill whole fishery products, clean water. It is to be made, handled and stored under conditions that protect it from contamination.*<br>***paragraph 5**. Steam used directly in contact with food is not to contain any substance that presents a hazard to health or is likely to contaminate the food.*<br>***paragraph 6.** Where heat treatment is applied to foodstuffs in hermetically sealed containers it is to be ensured that water used to cool the containers after heat treatment is not a source of contamination for the foodstuff.*		

areas, provided it does not contact edible product. For example, such reuse water may be used to move heavy solids, to flush the bottom of open evisceration troughs, or to wash antemortem areas, livestock pens, trucks, poultry cages, picker aprons, picking room floors, and similar areas within the establishment.				
6. *Water that does not meet the use conditions of paragraphs (g)(1) through (g)(5) of this section may not be used in areas where edible product is handled or prepared or in any manner that would allow it to adulterate edible product or create insanitary conditions.*	//	***Reg. (EC) 852/2004, Annex II, chapter VII, paragraph 2:*** *Where non-potable water is used, for example for fire control, steam production, refrigeration and other similar purposes, it is to circulate in a separate duly identified system. Non-potable water is not to connect with, or allow reflux into, potable water systems.*	//	***NO***
**h. Dressing rooms, lavatories, and toilets.**<br><br>1. *Dressing rooms, toilet rooms, and urinals must be sufficient in number, ample in size, conveniently located, and maintained in a sanitary condition and in good repair at all times to ensure cleanliness of all persons handling any product. They must be separate from the rooms and compartments in which products are processed, stored, or handled.*	//	***Reg. (EC) 852/2004, Annex II, chapter I, paragraph 3:*** *An adequate number of flush lavatories are to be available and connected to an effective drainage system. Lavatories are not to open directly into rooms in which food is handled.*<br><br>***Reg. (EC) 852/2004, Annex II, chapter I, paragraph 9:*** *Where necessary, adequate changing facilities for personnel are to be provided.*	***3.1 Infrastructure (building, equipment):*** *g) The specific clothes changing room(s) should be clean and ordered and, where possible, not used as a refectory or a smoking room. A separation between normal clothing, clean work clothing and used work clothing should be facilitated.*<br>*h) Toilets should not open directly to food handling areas. Preferably water flushing with use of foot/arm pedals should be present and reminders to wash hands and strategically placed signs informing about the obligation, when applicable, to remove protective clothing before using the toilets.*	***NO***
2. *Lavatories with running hot and cold water, soap, and towels, must be placed in or near toilet and urinal rooms and at such other places in the*	**FSIS-GD-2016-0003** *A handwashing lavatory shall be equipped to provide water at a temperature of at least 43°C (110°F)*	***Reg. (EC) 852/2004, Annex II, chapter I, paragraph 4:*** *An adequate number of washbasins is to be available, suitably located and designated for cleaning hands.*	***3.1 Infrastructure (building, equipment):*** *i) Hand washing facilities should be positioned conveniently between toilets/ changing rooms and the food*	***NO***

establishment as necessary to ensure cleanliness of all persons handling any product.	*through a mixing valve or combination faucet.*<br>*A steam mixing valve may not be used at a handwashing lavatory.*<br>*A self-closing, slow-closing, or metering faucet shall provide a flow of water for at least 15 seconds without the need to reactivate the faucet.*	*Washbasins for cleaning hands are to be provided with hot and cold running water, materials for cleaning hands and for hygienic drying. Where necessary, the facilities for washing food are to be separate from the hand-washing facility.*	*handling area, not excluding the possible need for additional wash hand basins in production areas near work stations; disinfectants, soap and towels for single use should be available; installations blowing warm air should only be present in rooms without food and non-hand-operable taps are desirable.*	
3. Refuse receptacles must be constructed and maintained in a manner that protects against the creation of insanitary conditions and the adulteration of product.	**FSIS-GD-2016-0003**<br>*Receptacles shall be durable, cleanable, insect and rodent resistant, leakproof, and nonabsorbent.*<br>*Plastic bags and wet strength paper bags may be used to line receptacles.*	***Reg. (EC) 852/2004, Annex II, chapter VI, paragraph 2:***<br>*Food waste, non-edible by-products and other refuse are to be deposited in closable containers, unless food business operators can demonstrate to the competent authority that other types of containers or evacuation systems used are appropriate. These containers are to be of an appropriate construction, kept in sound condition, be easy to clean and, where necessary, to disinfect.*<br>***Reg. (EC) 852/2004, Annex II, chapter VI, paragraph 4:***<br>*All waste is to be eliminated in a hygienic and environmentally friendly way in accordance with Community legislation applicable to that effect, and is not to constitute a direct or indirect source of contamination.*<br>***Reg. (EC) 853/2004 section V:***<br>*Food business operators must ensure that they: have equipment for washing hands used by staff handling exposed meat and products with taps designed to prevent the spread of contamination.*	*//*	***NO***

416.3 EQUIPMENT AND UTENSILS	***FSIS-GD-2016-0003***	***Reg (EC) 852/2004<br>Reg. (EC) 853/2004<br>Reg (UE) 2019/627<br>Reg. (EC) 1069/2009***	***Commission Notice 2022/C 355/01***	
a) Equipment and utensils used for processing or otherwise handling edible product or ingredients must be of such material and construction to facilitate thorough cleaning and to ensure that their use will not cause the adulteration of product during processing, handling, or storage. Equipment and utensils must be maintained in sanitary condition so as not to adulterate product.	**FSIS-GD-2016-0003**<br>*Materials that are used in the construction of utensils and food-contact surfaces of equipment may not allow the migration of deleterious substances or impart colors, odors, or tastes to food and under normal use conditions shall be:*<br>*a) Safe;*<br>*b) Durable, corrosion-resistant, and nonabsorbent;*<br>*c) Sufficient in weight and thickness to withstand repeated warewashing;*<br>*d) Finished to have a smooth, easily cleanable surface; and*<br>*e) Resistant to pitting, chipping, crazing, scratching, scoring, distortion, and decomposition.*<br>*Sponges may not be used in contact with cleaned and sanitized or in-use food-contact surfaces.*<br>*Wood and wood wicker may not be used as a food-contact surface, except hard maple or an equivalently hard, close-grained wood may be used for cutting boards; cutting blocks; bakers' tables; and utensils such as rolling pins, doughnut dowels, salad bowls, and chopsticks.*	***Reg (EC) 852/2004, Annex II, chapter II, paragraph 1:***<br>***f)*** *surfaces (including surfaces of equipment) in areas where foods are handled and in particular those in contact with food are to be maintained in a sound condition and be easy to clean and, where necessary, to disinfect. This will require the use of smooth, washable corrosion-resistant and non-toxic materials, unless food business operators can satisfy the competent authority that other materials used are appropriate.*<br>***Reg (EC) 852/2004, Annex II, chapter V, paragraph 1:***<br>*All articles, fittings and equipment with which food comes into contact are to:*<br>***a)*** *be effectively cleaned and, where necessary, disinfected. Cleaning and disinfection are to take place at a frequency sufficient to avoid any risk of contamination;*<br>***b)*** *be so constructed, be of such materials and be kept in such good order, repair and condition as to minimize any risk of contamination;*<br>***c)*** *with the exception of non-returnable containers and packaging, be so constructed, be of such materials and be kept in such good order, repair and condition as to enable them to be kept clean and, where necessary, to be disinfected; and*<br>***d)*** *be installed in such a manner as to allow adequate cleaning of the equipment and the surrounding area.*	***3.3 Pest control: emphasis on preventive activities***<br>*d) Unused equipment and rooms should be kept clean.*<br>***3.1 Infrastructure (building, equipment):***<br>*k) Equipment and monitoring/recording devices (e.g. thermometers) should be clean and the equipment suitable for contact with food products.*<br>*l) Attention should be paid to the different possibilities whereby the use of equipment can result in (cross-) contamination of food:*<br>*1. Prevention of contamination of the equipment by the environment e.g. condensation dripping from ceilings;*<br>*2. Prevention of contamination within the food handling equipment e.g. accumulation of food residues in slicing devices;*<br>*3. Prevention of contamination by raw materials: separate equipment (or cleaning and disinfection between uses) for raw products and cooked products (chopping boards, knives, dishes, clothing of staff, thermometers etc.).*	***NO***

		Reg (EC) 852/2004, Annex II, chapter V, paragraph 3:<br>*Where chemical additives have to be used to prevent corrosion of equipment and containers, they are to be used in accordance with good practice.*<br>***Reg. (EC) 853/2004, section V:***<br>*Food business operators must ensure that they:*<br>*5) have facilities for disinfecting tools with hot water supplied at not less than 82ºC, or an alternative system having an equivalent effect.*		
b) Equipment and utensils must not be constructed, located, or operated in a manner that prevents FSIS inspection program employees from inspecting the equipment or utensils to determine whether they are in sanitary condition.	//	***Reg (EC) 852/2004, Annex II Chapter V***<br><br>***Reg (EU) 2019/627, Article 3: Requirements subject to auditing, comma 1***	//	***NO***
c) Receptacles used for storing inedible material must be of such material and construction that their use will not result in the adulteration of any edible product or in the creation of insanitary conditions. Such receptacles must not be used for storing any edible product and must bear conspicuous and distinctive marking to identify permitted uses.	//	***Reg (EC) 852/2004, Annex II, chapter VI, paragraph 2:***<br>*Food waste, non-edible by-products and other refuse are to be deposited in closable containers, unless food business operators can demonstrate to the competent authority that other types of containers or evacuation systems used are appropriate. These containers are to be of an appropriate construction, kept in sound condition, be easy to clean and, where necessary, to disinfect.*<br>***Reg. (EC) 1069/2009, Article 4:***<br>*Member States shall ensure that an adequate system is in place on their territory ensuring that animal by-products are:*<br>*-collected, identified and transported without undue delay.*	***3. Waste management:***<br>*Compliance with the requirements in Chapter VI of Annex II to Regulation (EC) No 852/2004 can be best achieved and illustrated by the FBO by implementing procedures for each type of waste (animal by-products, spoiled food, chemical waste, redundant/used packing material). When applicable, it should be recorded who is responsible for the removal, how it is collected, where it is stored and how it is removed from the establishment.*	***NO***

416.4 SANITARY OPERATIONS	***FSIS-GD-2016-0003***	***Reg (EC) 852/2004***	***Commission Notice 2022/C 355/01***	
a) All food-contact surfaces, including food-contact surfaces of utensils and equipment, must be cleaned and sanitized as frequently as necessary to prevent the creation of insanitary conditions and the adulteration of product.	//	***Annex II, chapter V, paragraph 1:*** *All articles, fittings and equipment with which food comes into contact are to:* ***a)*** *be effectively cleaned and, where necessary, disinfected. Cleaning and disinfection are to take place at a frequency sufficient to avoid any risk of contamination.*	***3.2 Cleaning and disinfection:*** *a) What, when, how and by who to clean and disinfect should be considered.* *b) Typical steps should be the removal of visible dirt, followed by cleaning, followed by rinsing, followed by disinfection and rinsing again.* *c) Cleaning should start in high risk areas and should end in low risk areas. Materials and equipment for cleaning equipment should be different between low and high risk areas and in any case never move from a high contaminated area to a low one. Special attention must be paid to the contamination of disinfected surfaces due to splash when rinsing other surfaces.* *f) Visual checks on cleaning and sampling for analysis should be used to control disinfection activities.*	***NO*** ***Note:*** *In the context of US legislation, the term "cleaning and sanitizing" is always used, encompassing the concept of both cleaning and disinfection; in European legislation, on the other hand, the term "cleaning and, if necessary, disinfection" is used, thus leaving it up to the FBO to determine when it is appropriate to also proceed with disinfection.*
b) Non-food-contact surfaces of facilities, equipment, and utensils used in the operation of the establishment must be cleaned and sanitized as frequently as necessary to prevent the creation of insanitary conditions and the adulteration of product.	**FSIS-GD-2016-0003** *Non-food contact surfaces should be constructed of corrosion-resistant, nonabsorbent and smooth material.*	***Annex II, Chapter I,*** ***paragraph 1:*** *Food premises are to be kept clean and maintained in good repair and condition.* ***paragraph 2:*** *The layout, design, construction, siting and size of food premises are to:* ***a)*** *permit adequate maintenance, cleaning and/or disinfection, avoid or minimize air-borne contamination, and provide adequate working space to allow for the hygienic performance of all operations;* ***b)*** *be such as to protect against the accumulation of dirt, contact with toxic materials, the shedding of particles into food and the formation of condensation or undesirable mold on surfaces;* ***Annex II, chapter II, paragraph 1:***	//	***NO***

		In rooms where food is prepared, treated or processed (excluding dining areas and those premises specified in Chapter III, but including rooms contained in means of transport) the design and layout are to permit good food hygiene practices, including protection against contamination between and during operations…		
c) Cleaning compounds, sanitizing agents, processing aids, and other chemicals used by an establishment must be safe and effective under the conditions of use. Such chemicals must be used, handled, and stored in a manner that will not adulterate product or create insanitary conditions. Documentation substantiating the safety of a chemical's use in a food processing environment must be available to FSIS inspection program employees for review.	*//*	***Annex II, chapter I, paragraph 10:*** *Cleaning agents and disinfectants are not to be stored in areas where food is handled.* ***Annex II, chapter V, paragraph 1:*** *All articles, fittings and equipment with which food comes into contact are to:* ***a)*** *be effectively cleaned and, where necessary, disinfected. Cleaning and disinfection are to take place at a frequency sufficient to avoid any risk of contamination;* ***b)*** *be so constructed, be of such materials and be kept in such good order, repair and condition as to minimize any risk of contamination;* ***c)*** *with the exception of non-returnable containers and packaging, be so constructed, be of such materials and be kept in such good order, repair and condition as to enable them to be kept clean and, where necessary, to be disinfected; and* ***d)*** *be installed in such a manner as to allow adequate cleaning of the equipment and the surrounding area.* ***paragraph 3:*** *Where chemical additives have to be used to prevent corrosion of equipment and containers, they are to be used in accordance with good practice.*	***3.2 Cleaning and disinfection:*** *d) Potable water and/or cleaning agent or disinfectant should be used as much as needed to gain the desired effect in cleaning and/or disinfection. The water should be at an appropriate temperature and the chemicals should be used as per the manufacturer's instructions.* *e) Technical information should be available in your native language regarding detergents, disinfection agents (e.g. instructions for use, active component, contact time, concentration, use of potable water if appropriate).* ***3.6 Physical and chemical contaminations from production environment:*** *c) Only cleaning products suitable for food contact surfaces should be used in food processing environments where there is some possibility of incidental food contact. Other cleaning products should be only used outside periods of production.*	***NO***

d) Product must be protected from adulteration during processing, handling, storage, loading, and unloading at and during transportation from official establishments.	//	***Annex II, chapter IX, paragraph 3:*** *At all stages of production, processing and distribution, food is to be protected against any contamination likely to render the food unfit for human consumption, injurious to health or contaminated in such a way that it would be unreasonable to expect it to be consumed in that state.*	***3.6 Physical and chemical contaminations from production environment:*** *a) The frequency of the control of physical hazards (such as glass, plastic and metal) should be determined using a risk-based analysis (how big is the likelihood of occurrence in an establishment in question?).* *e) Possible chemical hazards should only be dealt with by specialized, trained staff.*	***NO***
416.5 EMPLOYEE HYGIENE	***FSIS-GD-2016-0003***	***Reg (EC) 852/2004***	***Commission Notice 2022/C 355/01***	
a) ***Cleanliness.*** *All persons working in contact with product, food-contact surfaces, and product-packaging materials must adhere to hygienic practices while on duty to prevent adulteration of product and the creation of insanitary conditions.*	**FSIS-GD-2016-0003** ***When****: Food employees shall clean their hands and exposed portions of their arms immediately before engaging in food preparation including working with exposed food, clean equipment and utensils, and unwrapped single-service and single-use articles and:* *- after touching bare human body parts other than clean hands and clean, exposed portions of arms;* *- after using the toilet room;* *- after coughing, sneezing, using a handkerchief or disposable tissue, using tobacco, eating, or drinking;* *- after handling soiled equipment or utensils;* *- during food preparation, as often as necessary to remove soil and contamination and to prevent cross contamination when changing tasks;* *- when switching between working with raw food and working with ready-to-eat food; and* *- after engaging in other activities that contaminate the hands.* ***Where:*** *Food employees shall clean their hands in a handwashing lavatory*	***Annex II, chapter VIII, paragraph 1:*** *Every person working in a food-handling area is to maintain a high degree of personal cleanliness and is to wear suitable, clean and, where necessary, protective clothing.*	***3.11 Personal (hygiene, health status):*** *b) Hands should be washed regularly (and disinfected if necessary), as a minimum, before starting work, after using the lavatory, after breaks, after rubbish disposal, after coughing or sneezing (in a disposable paper or, if no alternative, into your elbow), after handling of raw materials, between tasks, etc. Disposable gloves used hygienically can be effective in preventing cross contamination when handling ready-to-eat foods. Hands must be washed thoroughly before and after use. Gloves must be used only once and should be changed between tasks to prevent cross contamination.*	***NO***

	or approved automatic handwashing facility and may not clean their hands in a sink used for food preparation, or in a service sink or a curbed cleaning facility used for the disposal of mop water and similar liquid waste.<br>***Fingernails:** Food employees shall keep their fingernails trimmed, filed, and maintained so the edges and surfaces are cleanable and not rough. Unless wearing intact gloves in good repair, a food employee may not wear fingernail polish or artificial fingernails when working with exposed food.*<br>***Jewelry:** While preparing food, food employees may not wear jewelry on their arms and hands. This section does not apply to a plain ring such as a wedding band.*			
*b) **Clothing.** Aprons, frocks, and other outer clothing worn by persons who handle product must be of material that is disposable or readily cleaned. Clean garments must be worn at the start of each working day and garments must be changed during the day as often as necessary to prevent adulteration of product and the creation of insanitary conditions.*	*//*	***Annex II, chapter VIII, paragraph 1:***<br>*Every person working in a food-handling area is to maintain a high degree of personal cleanliness and is to wear suitable, clean and, where necessary, protective clothing.*	***3.11 Personal (hygiene, health status):***<br>*c) Hair covers (and beard snoods) should be considered and appropriate clothing with high degree of cleanliness, minimum of pockets, absence of jewelry and watches. The use by workers of clothing or items of clothing with different colors is recommended in different microbiological risk areas.*<br>*d) Protective clothing should preferably not be worn when using the toilets or when wheeling the rubbish bins onto the street.*<br>*f) First aid kits should be easily accessible and available for immediate use.*<br>*g) The number of visitors should be minimized and visits should follow the conditions set by the FBO so as not to compromise the food safety. Visitors should at least wash hands and wear appropriate protective clothing, provided by the FBO.*	***NO***

*c) **Disease control.** Any person who has or appears to have an infectious disease, open lesion, including boils, sores, or infected wounds, or any other abnormal source of microbial contamination, must be excluded from any operations which could result in product adulteration and the creation of insanitary conditions until the condition is corrected.*	//	***Annex II, chapter VIII, paragraph 2:***<br>*No person suffering from, or being a carrier of a disease likely to be transmitted through food or afflicted, for example, with infected wounds, skin infections, sores or diarrhea is to be permitted to handle food or enter any food-handling area in any capacity if there is any likelihood of direct or indirect contamination. Any person so affected and employed in a food business and who is likely to come into contact with food is to report immediately the illness or symptoms, and if possible their causes, to the food business operator.*	***3.11 Personal (hygiene, health status):***<br>*a) Personnel should be aware of hazards from gastro-intestinal infections, hepatitis and wounds with appropriate exclusion from food handling or suitable protection; relevant health problems should be reported to the manager. Special consideration should be given to temporary workers who might be less familiar with potential hazards.*	***NO***
416.6 TAGGING INSANITARY EQUIPMENT, UTENSILS, ROOMS OR COMPARTMENTS		***Reg. (EU) 2017/625***		
When an FSIS program employee finds that any equipment, utensil, room, or compartment at an official establishment is insanitary or that its use could cause the adulteration of product, he will attach to it a "U.S. Rejected" tag. Equipment, utensils, rooms, or compartments so tagged cannot be used until made acceptable. Only an FSIS program employee may remove a "U.S. Rejected" tag.	//	***Title VII, chapter I, Article 138: Actions in the event of established non-compliance***<br>*1. Where the non-compliance is established, the competent authorities shall take:*<br>*a) any action necessary to determine the origin and extent of the non-compliance and to establish the operator's responsibilities; and*<br>*b) appropriate measures to ensure that the operator concerned remedies the non-compliance and prevents further occurrences of such non-compliance.*	//	***NO***

**9 CFR part 416<br>SSOP**	**USA GUIDELINES DIRECTIVES and other supplementary documents**<br>• ***FSIS-GD-2020-0009***<br>• ***FSIS DIRECTIVE 5000.1 (REV.8)***<br>• ***FSIS-DIRECTIVE 5000.4 (REV.3)***<br>• ***Sanitation Standard Operating Procedures (2019)***	**EU LEGISLATION**<br>• ***Regulation (EC) 852/2004***<br>• ***Regulation (EU) 2017/625***<br>• ***Regulation (EU) 2019/627***	**EU SUPPLEMENTARY DOCUMENTS**<br>• ***Commission Notice 2022/C 355/01***	**IMPLEMENTATION?**
416.11 GENERAL RULES	***FSIS-GD-2020-0009 Sanitation Standard Operating Procedures (2019)***	***Reg. (EC) 852/2004***		
Each official establishment shall develop, implement, and maintain written standard operating procedures for sanitation (Sanitation SOP's) in accordance with the requirements of this part.	*The Sanitation SOPs cover* ***the entire establishment*** *and* ***all shifts*** *of operation.*<br><br>***Sanitation Standard Operating Procedures (2019)***<br>*After completion of this module, the participant will be able to:*<br>*1. Explain the meaning and significance of the following terms:*<br>*a. Sanitation SOP*<br>*b. Responsible person*<br>*c. Regulatory control action*<br>*d. Pre-operational sanitation procedures*<br>*e. Operational sanitation procedures*<br>*f. Sanitation SOP Implementation & Monitoring*<br>*g. Sanitation SOP Maintenance*<br>*h. Sanitation SOP Corrective Actions*<br>*i. Sanitation SOP Recordkeeping.*	***Article 4, paragraph 3(b)***<br>*Food business operators shall, as appropriate, adopt the following specific hygiene measures:*<br>*b) procedures necessary to meet targets set to achieve the objectives of this Regulation.*	*//*	***YES***<br><br>***Note:*** *European legislation does not require the development of sanitization standard operating procedures (SSOPs). In Europe they are referred to as GHPs, which are equivalent to the sanitation requirements of SPSs. Instead, SSOPs are a step up from SPSs and are intended to monitor the effectiveness of cleaning and disinfection procedures conducted before and during operations in order to work with clean and sanitized surfaces and prevent contamination and adulteration of products. For more information FSIS developed a SSOP model (FSIS-GD-2020-0009) and a training module (Sanitation Standard Operating Procedures (2019)).*

416.12 DEVELOPMENT OF SSOP	***FSIS-DIRECTIVE 5000.4 (REV.3)***	***Reg. (EC) 852/2004***		
a) The Sanitation SOP's shall describe all procedures an official establishment will conduct daily, before and during operations, sufficient to prevent direct contamination or adulteration of product(s).	*//*	***Reg. (EC) 852/2004 Annex II, chapter I***<br>*General requirements for food premises.*<br>***Annex II, chapter II***<br>*Specific requirements in rooms where foodstuffs are prepared, treated or processed.*<br>***Annex II, chapter V***<br>*Equipment requirements.*<br>***Annex II, chapter IX***<br>*Provisions applicable to foodstuffs.*	*//*	***YES***
b) The Sanitation SOP's shall be signed and dated by the individual with overall authority on-site or a higher level official of the establishment. This signature shall signify that the establishment will implement the Sanitation SOP's as specified and will maintain the Sanitation SOP's in accordance with the requirements of this part. The Sanitation SOP's shall be signed and dated upon initially implementing the Sanitation SOP's and upon any modification to the Sanitation SOP's.	*//*	***Reg. (EC) 852/2004***<br>***Article 1, paragraph 1, (a) and (d)***<br>*This Regulation lays down general rules for food business operators on the hygiene of foodstuffs, taking particular account of the following principles:*<br>*a) primary responsibility for food safety rests with the food business operator;*<br>*d) general implementation of procedures based on the HACCP principles, together with the application of good hygiene practice, should reinforce food business operators' responsibility.*<br>***Reg. (EC) 852/2004,***<br>***Annex II, chapter 11bis, paragraph 2 (a), (b), (c)***<br>*Management commitment shall include:*<br>*a) ensuring that roles and responsibilities are clearly communicated within each activity of the food business;*<br>*b) maintaining the integrity of the food hygiene system when changes are planned and implemented;*<br>*c) verifying that controls are being performed timely and*	*//*	***YES***

		efficiently and documentation is up to date.		
c) Procedures in the Sanitation SOP's that are to be conducted prior to operations shall be identified as such, and shall address, at a minimum, the cleaning of food contact surfaces of facilities, equipment, and utensils.	***FSIS-DIRECTIVE 5000.4 (REV. 3)*** *Many establishments incorporate the cleaning of non-food contact surfaces into the Sanitation SOPs and identify these procedures as pre-op sanitation procedures (e.g., for walls, floors, etc.). In cases like this, if IPP observe an unclean or insanitary non-food contact surface that has been identified by the establishment as an area covered by the pre-op sanitation procedure in its Sanitation SOP plan, IPP are to cite noncompliance with 9 CFR 416.13 because the establishment failed to implement or monitor a pre-op sanitation procedure.*	***Reg. (EC) 852/2004*** ***Annex II, chapter I*** *General requirements for food premises.* ***Annex II, chapter II*** *Specific requirements in rooms where foodstuffs are prepared, treated or processed.* ***Annex II, chapter V*** *Equipment requirements.* ***Annex II, chapter IX*** *Provisions applicable to foodstuffs.*	//	***YES***
d) The Sanitation SOP's shall specify the frequency with which each procedure in the Sanitation SOP's is to be conducted and identify the establishment employee(s) responsible for the implementation and maintenance of such procedure(s).	//	***Reg. (EC) 852/2004*** ***Annex II, chapter V, paragraph 1 (a)*** *All articles, fittings and equipment with which food comes into contact are to be effectively cleaned and, where necessary, disinfected. Cleaning and disinfection are to take place at a frequency sufficient to avoid any risk of contamination.*	//	***YES***
416.13 IMPLEMENTATION OF SSOP	***FSIS DIRECTIVE 5000.1 (REV.8)***	***Reg. (EC) 852/2004***		
a) Each official establishment shall conduct the pre-operational procedures in the Sanitation SOP's before the start of operations.	//	***Annex II, chapter II, paragraph 1*** *In rooms where food is prepared, treated or processed the design and layout are to permit good food hygiene practices, including protection against contamination between and during operations.*	//	***YES***
b) Each official establishment shall conduct all other procedures in the Sanitation SOP's at the frequencies specified.	//	***Annex II, chapter V paragraph 1 (a)*** *All articles, fittings and equipment with which food comes into contact are to:*	//	***YES***

		a) be effectively cleaned and, where necessary, disinfected. Cleaning and disinfection are to take place at a frequency sufficient to avoid any risk of contamination.		
c) Each official establishment shall monitor daily the implementation of the procedures in the Sanitation SOP's.	***FSIS DIRECTIVE 5000.1 (REV.8)***<br>*If environmental sampling is included in the Sanitation SOP, IPP are to verify that the establishment is following those procedures. IPP are to observe the establishment collecting samples, review sample results, and verify that the establishment takes corrective actions specified in the Sanitation SOP for results that do not meet the specified criteria.*	***Article 4, paragraph 3 (b)***<br>*Food business operators shall, as appropriate, adopt the following specific hygiene measures:*<br>*b) procedures necessary to meet targets set to achieve the objectives of this Regulation.*	*//*	***YES***
416.14 MAINTENANCE OF SSOP	***FSIS DIRECTIVE 5000.1 (REV.8)***	***Reg. (EC) 852/2004***		
Each official establishment shall routinely evaluate the effectiveness of the Sanitation SOP's and the procedures therein in preventing direct contamination or adulteration of product(s) and shall revise both as necessary to keep them effective and current with respect to changes in facilities, equipment, utensils, operations, or personnel.	***FSIS DIRECTIVE 5000.1 (REV.8)***<br>*Construction and removal of walls, ceilings, and floors may cause harborage sites for L. monocytogenes (Lm) to be dislodged from otherwise protected areas.*	***Reg. (EC) 852/2004,***<br>***Article 5, paragraph 2***<br>*When any modification is made in the product, process, or any step, food business operators shall review the procedure and make the necessary changes to it.*<br>***f)*** *establishing procedures, which shall be carried out regularly, to verify that the measures outlined in subparagraphs (a) to (e) are working effectively;*<br>***Article 5, paragraph 4 (b)***<br>*Food business operators shall:*<br>*b) ensure that any documents describing the procedures developed in accordance with this Article are up-to-date at all times.*<br>***Annex II, chapter I***<br>*General requirements for food premises.*<br>***Annex II, chapter II***	*//*	***YES***

		Specific requirements in rooms where foodstuffs are prepared, treated or processed.<br>***Annex II, chapter V***<br>*Equipment requirements.*		
416.15 CORRECTIVE ACTIONS	***FSIS-GD-2020-0009***	***Reg. (EC) 852/2004***		
a) Each official establishment shall take appropriate corrective action(s) when either the establishment or FSIS determines that the establishment's Sanitation SOP's or the procedures specified therein, or the implementation or maintenance of the Sanitation SOP's, may have failed to prevent direct contamination or adulteration of product(s).	*//*	***Article 4, paragraph 3(b)***<br>*Food business operators shall, as appropriate, adopt the following specific hygiene measures:*<br>*b) procedures necessary to meet targets set to achieve the objectives of this Regulation.*	*//*	***YES***<br><br>***Note:*** *According to US legislation, failure to record a pre-operational SSOP considers the product adulterated therefore corrective actions must be implemented not only on the process but also on the product processed on an uncontrolled surface; in the case of an operational SSOP the concept is the same. In recording corrective actions this should always be made explicit.*
b) Corrective actions include procedures to ensure appropriate disposition of product(s) that may be contaminated, restore sanitary conditions, and prevent the recurrence of direct contamination or adulteration of product(s), including appropriate reevaluation and modification of the Sanitation SOP's and the procedures specified therein or appropriate improvements in the execution of the Sanitation SOP's or the procedures specified therein.	***FSIS-GD-2020-0009:***<br>*The document provides an explanatory model of a form for monitoring procedures and implementing corrective actions.*	***Article 5, paragraph 2(e)***<br>*e) establishing corrective actions when monitoring indicates that a critical control point is not under control.*	*//*	***YES***
416.16 RECORDKEEPING REQUIREMENTS		***Reg. (EC) 852/2004***	***Commission Notice 2022/C 355/01***	
a) Each official establishment shall maintain daily records sufficient to document the implementation and monitoring of the Sanitation SOP's and any corrective actions taken. The establishment employee(s) specified	*//*	***Article 5, paragraph 2(g)***<br>*g) establishing documents and records commensurate with the nature and size of the food business to demonstrate the effective application of the measures.*	*//*	***YES***

in the Sanitation SOP's as being responsible for the implementation and monitoring of the procedure(s) specified in the Sanitation SOP's shall authenticate these records with his or her initials and the date.		***Article 5, paragraph 4***<br>*Food business operators shall:*<br>*b) ensure that any documents describing the procedures developed in accordance with this Article are up-to-date at all times;*<br>*c) retain any other documents and records for an appropriate period.*		
b) Records required by this part may be maintained on computers provided the establishment implements appropriate controls to ensure the integrity of the electronic data.	//	//	***Commission Notice 2022/C 355/01***<br>*Records can be kept electronically as long as they can be made available to the competent authorities at their request e.g. during an audit, to verify the effective application of the requirements.*	***YES***
c) Records required by this part shall be maintained for at least 6 months and made available to FSIS. All such records shall be maintained at the official establishment for 48 hours following completion, after which they may be maintained off-site provided such records can be made available to FSIS within 24 hours of request.	//	***Article 5, paragraph 4***<br>*Food business operators shall:*<br>*c) retain any other documents and records for an appropriate period.*	***11- Documentation and Record keeping***<br>*Records should be kept for an appropriate period of time in any format. That period should be long enough to ensure information to be available in case of an alert that can be traced back to the food in question.*	***YES***
416.17 AGENCY VERIFICATION	***FSIS-DIRECTIVE 5000.1 (Rev.8)***<br>***FSIS-DIRECTIVE 5000.4 (Rev.3)***	***Reg. (EU) 2017/625***<br>***Reg. (EU) 2019/627***		
FSIS shall verify the adequacy and effectiveness of the Sanitation SOP's and the procedures specified therein by determining that they meet the requirements of this part. Such verification may include:<br>*a) reviewing the Sanitation SOP's;*<br>*b) reviewing the daily records documenting the implementation of the Sanitation SOP's and the procedures specified therein*	***FSIS-DIRECTIVE-5000.1 (Rev.8):***<br>*IPP's primary role is to use their findings to determine whether or not the establishment is implementing Sanitation SOPs effectively to prevent contamination or adulteration of products. IPP are to perform two general types of Sanitation SOP verification tasks to verify that an establishment is meeting the regulatory requirements for Sanitation SOPs. Each type*	***Reg. (EU) 2017/625 Article 10, chapter 1 (c):***<br>*c) The competent authorities shall perform official controls on operators as regards activities, including the keeping of animals, equipment, means of transport, premises and other places under their control and their surroundings and on related documentation.*<br>***Article 14***<br>*Methods and techniques for official controls.*<br>***Article 15***	//	***YES***

and any corrective actions taken or required to be taken;<br>c) *direct observation of the implementation of the Sanitation SOP's and the procedures specified therein and any corrective actions taken or required to be taken; and*<br><br>d) *direct observation or testing to assess the sanitary conditions in the establishment.*	*includes a* recordkeeping verification *task and a review and observation (e.g., "hands-on") task. The general types of Sanitation SOP tasks are:*<br>*1. <u>Pre-Operational Sanitation SOP Verification</u>: IPP are to use the 'Pre-Op Records Review' and 'Pre- Op Review and Observation' tasks to verify that the establishment implements the pre-operational procedures in the Sanitation SOP effectively to prevent contamination of food contact surfaces or adulteration of products prior to operations. IPP are to verify that the establishment meets all Sanitation SOP regulatory requirements (monitoring, maintenance, corrective action).*<br>*2. <u>Operational Sanitation SOP Verification</u>: IPP are to use the 'Operational SSOP Records Review' and 'Operational SSOP Review and Observations' tasks to verify that the establishment implements the operational procedures in the Sanitation SOP effectively to prevent contamination of food contact surfaces or adulteration of products during operations. IPP will verify that the establishment meets all Sanitation SOP regulatory requirements (monitoring, recordkeeping, maintenance, corrective action).*<br>*IPP are to review the establishment's written Sanitation SOPs in preparation to verify pre-operational and operational Sanitation SOP requirements. IPP are to be familiar with the procedures used and the*	*Obligations of operators.*<br>***Commission Implementing Regulation (EU) 2019/627, Article 3:***<br>*When auditing good hygiene practices in establishments, the competent authorities shall verify that food business operators handling products of animal origin apply procedures continuously and properly concerning at least the following:*<br>*a) the design and maintenance of premises and equipment;*<br>*b) pre-operational, operational and post-operational hygiene;*<br>*c) personal hygiene;*<br>*d) training in hygiene and in work procedures;*<br>*e) pest control;*<br>*f) water quality;*<br>*g) temperature control; h) controls on animals or food entering and leaving the establishment, and any accompanying documentation.*	*//*	***YES***

	monitoring procedures and frequencies specified in the Sanitation SOPs. If IPP are familiar with the program, this review serves as a way to ensure that there have been no modifications from the last time IPP performed the task. When IPP are not familiar with the written Sanitation SOP or they are aware the program has been changed, IPP are to verify compliance with 9 CFR 416.12. If environmental sampling is included in the Sanitation SOP, IPP are to verify that the establishment is following those procedures. IPP are to observe the establishment collecting samples, review sample results, and verify that the establishment takes corrective actions specified in the Sanitation SOP for results that do not meet the specified criteria. IPP are to complete this verification as part of the applicable Sanitation SOP verification task.<br>***FSIS-DIRECTIVE-5000.4 (REV.3)***<br>*This directive provides instructions to inspection program personnel (IPP) regarding how to perform the Public Health Information System (PHIS) Pre-Operational (Pre-Op) Sanitation Standard Operating Procedures (Sanitation SOP) verification task in meat and poultry slaughter and processing operations, and official import establishments.*			

**9 CFR part 417 HACCP**	**USA GUIDELINES DIRECTIVES and other supplementary documents**	**EU LEGISLATION**	**EU SUPPLEMENTARY DOCUMENTS**	**IMPLEMENTATION?**
	• ***FSIS-GD-2018-0005***<br>• ***FSIS-GD-2020-0008***<br>• ***FSIS-GD-2021-0010***<br>• ***FSIS Directive 7530.1 (REV.4)***<br>• ***FSIS Directive 7530.2 (REV.1)***<br>• ***Microbiology of thermally processed commercially sterile and shelf-stable meat and poultry product (2005)***<br>• ***Thermally Processed Products FSA Tool VS3 (2020)***<br>• ***FSIS Thermal Processing/Commercially Sterile Self-Paced Training Course (2021)***<br>+<br>*9 CFR part 431*	• ***Regulation (EC) 178/2002***<br>• ***Regulation (EC) 852/2004***<br>• ***Regulation (EC) 853/2004***<br>• ***Regulation (EC) 2073/2005***<br>• ***Regulation (EU) 2017/625***<br>• ***Regulation (EU) 2021/382***	• ***Commission Notice 2022/C 355/01***<br>• ***Opinion of the Scientific Panel on Biological Hazards on the request from the Commission related to Clostridium spp. in foodstuffs***	
**417.1 *DEFINITIONS***	***FSIS-GD-2020-0008 FSIS Directive 7530.1 (REV.4)***	***Reg. (EC) 178/2002 Reg. (EU) 2017/625***	***Commission Notice 2022/C 355/01***	
For purposes of this part, the following definitions shall apply:<br>***1. Corrective action:***<br>*Procedures to be followed when a deviation occurs.*	*Definitions*<br>***Corrective action:***<br>*Action to be taken when a **deviation** or unforeseen hazard occurs.*	//	*Definitions*<br>***Corrective action:***<br>*Any action taken when a deviation occurs in order to re-establish control, segregate and determine the disposition of the*	***NO***<br>***Note:** specific definitions related to the implementation of the HACCP system (CCP, corrective*

	Deviation:<br>*Failure to meet a critical limit.*<br><br>***FSIS Directive 7530.1 (REV.4)***<br>*Handling a process deviation or abnormal container of thermally processed, commercially sterile canned product.*<br>*Attachment: causes of process deviations.*		*affected product if any and prevent or minimize reoccurrence of the deviation. (This definition is taken from General Principles of Food Hygiene CXC 1-1969).*	*actions, critical limits) are not reported in the EU legislation.*
3. Critical limit:<br>*The maximum or minimum value to which a physical, biological, or chemical hazard must be controlled at a critical control point to prevent, eliminate, or reduce to an acceptable level the occurrence of the identified food safety hazard.*	*//*	*//*	***Critical limit:***<br>*A criterion, observable or measurable, relating to a control measure at a CCP which separates acceptability from unacceptability of the food (as above).*	***NO***
4. Food safety hazard:<br>*Any biological, chemical, or physical property that may cause a food to be unsafe for human consumption.*	***Food safety:***<br>*Handling, preparing, and storing food in a way that best reduces the risk of individuals becoming sick from foodborne illnesses.*	***Reg. (EC) 178/2002, Article 3:***<br>***Hazard:***<br>*A biological, chemical or physical agent in, or condition of, food or feed with the potential to cause an adverse health effect.*<br>***Risk:***<br>*A function of the probability of an adverse health effect and the severity of that effect, consequential to a hazard.*<br>***Reg. (EU) 2017/625, Article 3:***<br>***Hazard:***<br>*Any agent or condition with the potential to have an adverse effect on human, animal or plant health, animal welfare or the environment.*<br>***Risk**:*<br>*A function of the probability of an adverse effect on human, animal or plant health, animal welfare or the environment and of the severity of that effect, consequential to a hazard.*	***Hazard:***<br>*A biological (e.g. Salmonella), chemical (e.g. dioxin, allergens) or physical (e.g. hard, sharp foreign bodies as pieces of glass, metal) agent in food with the potential to cause an adverse health effect (as above + examples).*<br>***Significant hazard:***<br>*A hazard identified by a hazard analysis, as reasonably likely to occur at an unacceptable level in the absence of control, and for which control is essential given the intended use of the food (as above).*	***NO***

5. HACCP System:<br>*The HACCP plan in operation, including the HACCP plan itself.*	***HACCP system:*** *The HACCP system is defined as the HACCP plan in operation, including the HACCP plan itself. The HACCP plan in operation includes the hazard analysis, any supporting documentation including prerequisite programs supporting decisions in the hazard analysis, and all HACCP records.*	//	***HACCP-based procedures or 'HACCP':***<br>*Procedures based on the hazard analysis and critical control points (HACCP) principles i.e. an own-check system which identifies, evaluates and controls hazards which are significant for food safety consistent with the HACCP principles.*<br>***HACCP plan:***<br>*Documentation or set of documents, prepared in accordance with the principles of HACCP to ensure control of significant hazards in the food business, available in any format. The initial HACCP plan shall be updated if there are changes in the production and must be supplemented with records from outcomes of monitoring and verification, and from corrective actions taken.*	***NO***
6. Hazard:<br>*See: Food Safety Hazard.*	//	***Hazard:***<br>*See above*	***Hazard:***<br>*See above*	***NO***
7. Preventive measure:<br>*Physical, chemical, or other means that can be used to control an identified food safety hazard.*	***Preventive measure:***<br>*Physical, chemical, or other means that the establishment can use to control food safety hazards reasonably likely to occur in the production process.*	//	//	***NO***
8. Process-monitoring instrument:<br>*An instrument or device used to indicate conditions during processing at a critical control point.*	//	//	//	***NO***
9. Responsible establishment official:<br>*The individual with overall authority on-site or a higher level official of the establishment.*	//	***Reg. (EC) 178/2002, Article 3:***<br>***Food business operator (FBO):***<br>*Natural or legal persons responsible for ensuring that the requirements of food law are met within the food business under their control.*<br>***Reg. (EU) 2017/625, Article 3, Operator:***<br>*Any natural or legal person subject to one or more of the obligations*	//	***NO***<br>***Note:*** *Under 417.1, the responsible establishment official does not necessarily coincide with the Food Business Operator (as defined by European legislation), but Is a figure with the necessary skills to determine how HACCP should be implementing, ensuring that the establishment implements it accordingly.*

		provided for in the rules referred to in Article 1(2).		
417.2 HAZARD ANALYSIS AND HACCP PLAN	***FSIS-GD-2018-0005<br>FSIS-GD-2020-0008<br>FSIS-GD-2021-0010<br>FSIS Directive 7530.1 (REV.4)<br>FSIS Directive 7530.2 (REV. 1)<br>Microbiology of thermally processed commercially sterile and shelf-stable meat and poultry product (2005).<br>Thermally Processed Products FSA Tool VS3 (2020).<br>FSIS Thermal Processing/ Commercially Sterile Self-Paced Training Course (2021).<br>+<br>9 CFR part 431***	***Reg. (EC) 852/2004<br>Reg. (EC) 853/2004<br>Reg. (EC) 2073/2005<br>Reg. (EU) 2017/625***	***Commission Notice 2022/C 355/01<br>Opinion of the Scientific Panel on Biological Hazards on the request from the Commission related to Clostridium spp. in foodstuffs***	
**a) Hazard analysis**<br>**1.** *Every official establishment shall conduct, or have conducted for it, a hazard analysis to determine the food safety hazards reasonably likely to occur in the production process and identify the preventive measures the establishment can apply to control those hazards. The hazard analysis shall include food safety hazards that can occur before, during, and after entry into the establishment. A food safety hazard that is reasonably likely to occur is one for which a prudent establishment would establish controls because it historically has occurred, or because there*	*//*	***Reg. (EC) 852/2004, Article 5: Hazard analysis and critical control points****.*<br>***Paragraph 1****:*<br>*Food business operators shall put in place, implement and maintain a permanent procedure or procedures based on the HACCP principles.*	*HACCP-based procedures are mandatory for all food business operators except primary producers in accordance with Article 5 of Regulation (EC) 852/2004. HACCP-based procedures represent a systematic approach to the identification, evaluation and control of food safety hazards e.g. biological, chemical (including allergens) and physical hazards.*<br>*HACCP-based procedures are considered to be a useful tool for food business operators to identify and control hazards that may occur in food and during food processing in their own establishment. The HACCP-based procedures should be science/risk-based and systematic, identifying significant hazards at each step of the production chain, and measures for control of those hazards, to ensure the safety of food.*	***NO***

is a reasonable possibility that it will occur in the particular type of product being processed, in the absence of those controls.			*HACCP-based procedures are tools to identify and assess hazards and establish control systems that focus on prevention, as opposed to older systems that relied mainly on end product testing.*<br>*Prior to the application of the HACCP-based procedures to any business, the food business operator should have implemented the PRP, including GHP and the other measures laid down in Regulation (EC) No 178/2002.*	
2. *A flow chart describing the steps of each process and product flow in the establishment shall be prepared, and the intended use or consumers of the finished product shall be identified.*	***FSIS-GD-2020-0008***<br>*Preliminary Steps:*<br>*a) assemble the HACCP team;*<br>*b) describe the food and methods of producing and distributing the product;*<br>*c) develop and verify process flow charts;*<br>*d) decide how products can be grouped using the process categories in 9 CFR 417.2(b)(1).*	*//*	*The preliminary activities below are not explicitly laid down in EU legislation, nevertheless they are considered as essential when developing and implementing HACCP-based procedures. These preliminary activities traditionally consist of 5 steps and when combines with the 7 HACCP principles, result a 12-steps approach:*<br>*1. Assembly of a multidisciplinary HACCP team;*<br>*2. Description of the product(s) at the end of process (called hereafter 'end product');*<br>*3. Identification of intended use;*<br>*4. Construction of a flow diagram (description of manufacturing process);*<br>*5. On-site confirmation of flow diagram.*	***NO***
3. *Food safety hazards might be expected to arise from the following:*<br>*(i) Natural toxins;*<br>*(ii) Microbiological contamination;*<br>*(iii) Chemical contamination;*<br>*(iv) Pesticides;*<br>*(v) Drug residues;*<br>*(vi) Zoonotic diseases;*<br>*(vii) Decomposition;*<br>*(viii) Parasites;*<br>*(ix) Unapproved use of direct or indirect food or color additives; and (x) Physical hazards.*	*//*	*//*	*//*	***NO***

**b) The HACCP plan**<br>***1.*** *Every establishment shall develop and implement a written HACCP plan covering each product produced by that establishment whenever a hazard analysis reveals one or more food safety hazards that are reasonably likely to occur, based on the hazard analysis conducted in accordance with paragraph (a) of this section, including products in the following processing categories:*<br>*(i) Slaughter—all species.*<br>*(ii) Raw product—ground.*<br>*(iii) Raw product—not ground.*<br>*(iv) Thermally processed—commercially sterile.*<br>*(v) Not heat treated—shelf stable.*<br>*(vi) Heat treated—shelf stable.*<br>*(vii) Fully cooked—not shelf stable.*<br>*(viii) Heat treated but not fully cooked—not shelf stable.*<br>*(ix) Product with secondary inhibitors—not shelf stable.*	***FSIS-GD-2020-0008***<br>*The decision about processing categories completes the preliminary steps that prepare you to develop a HACCP system.*	***Reg. (EC) 852/2004, Article 5: Hazard analysis and critical control points.***<br>***paragraph 1****:*<br>*Food business operators shall put in place, implement and maintain a permanent procedure or procedures based on the HACCP principles.*<br>***Reg. (EC) 852/2004, Article 5, paragraph 3:***<br>*Paragraph 1 shall apply only to food business operators carrying out any stage of production, processing and distribution of food after primary production and those associated operations listed in Annex I.*	//	***YES***<br><br>***Note:*** *European legislation does not require prior selection of the processing category.*
2. *A single HACCP plan may encompass multiple products within a single processing category identified in this paragraph, if the food safety hazards, critical control points, critical limits, and procedures required to be identified and performed in paragraph (c) of this section are essentially the same, provided that any required features of the plan that are unique to a specific product are clearly delineated in the plan and are observed in practice.*	***FSIS-GD-2020-0008***<br>*The HACCP system may control all products in the same process category using a single HACCP plan, if the processes are essentially the same. That is advantageous for very small establishments that produce several different products. If those products differ only in characteristics that would not affect safety – such as the amount or kind of seasoning used, such as, hot vs. mild – and if the food safety hazards, critical control points, critical limits, and procedures listed in*	//	***3.2 Simplified HACCP-based procedures:***<br>*Similar products can be grouped together for the implementation of procedures based on the HACCP principles if they are produced in the same way and share common hazards.*	***NO***

	9 CFR 417.2 (c) are the same, then those products are in the same process category and may be covered by the same HACCP plan (9 CFR 417.2 (b)(2)).			
3. *HACCP plans for thermally processed/commercially sterile products do not have to address the food safety hazards associated with microbiological contamination if the product is produced in accordance with the requirements of part 431 of this chapter.*	***FSIS-GD-2021-0010*** *Processors of Thermally processed, commercially sterile (TPCS) products must identify their biological, physical, and chemical food safety hazards when performing their hazard analysis.* *Under 9 CFR 417.2(b)(3) of the HACCP regulations, establishments do not have to address the microbiological food safety hazards identified in its hazard analysis if the product is produced in accordance with the requirements of 9 CFR Part 431*. However, canning establishments that identify chemical or physical food safety hazards as reasonably likely to occur (RLTO) are to address those hazards in their HACCP plan. The regulations provide that canning establishments do not have to address microbiological hazards in their HACCP plan because FSIS recognized that the canning regulations were based on HACCP concepts and provide for the analysis of thermal processing systems and controls to exclude biological food safety hazards. However, a canning establishment may choose to address the microbiological food safety hazards in its HACCP plan**. In either case, the requirements in 9 CFR parts 431 and 417*	***Reg. (EC) 852/2004, Article 5, Paragraph 2:*** *The HACCP principles referred to in paragraph 1 consist of the following:* *identifying any hazards that must be prevented, eliminated or reduced to acceptable levels;* <br>***Reg. (EC) 2073/2005*** *on microbiological criteria for foodstuffs.*	*//*	***NO*** <br>***Notes:*** *European legislation does not provide that establishments may not include microbiological hazards within the HACCP plan. In fact, they are required to identify any hazard, including microbiological hazards within the HACCP plan independently of the product. In addition, there is no predefined HACCP model for thermally processed commercially sterile products.* <br>***Note 2****: Reg. (EC) 2073/2005 Does not establish safety and/or hygiene criteria for Clostridium spp.*

	must be met through the establishment's HACCP system.<br><br>****9 CFR part 431 Thermally processed, commercially sterile products.** It defines the technical and procedural requirements for the proper production and marketing of these products.*<br>*low acid product: canned products in which any component ha a pH value above 4.6.*<br><br>***** Microbiology of thermally processed commercially sterile and shelf-stable meat and poultry product (2005).** For low-acid products as the product under study, it specifies thermal processing standards targeting Clostridium botulinum and Clostridium sporogenes*<br>*12-log reduction of C. botulinum and 5-log reduction of C. sporogenes.*<br><br>***FSIS-GD-2018-0005***<br>*Process steps, potential hazards, and frequently used controls: processing.*<br><br>***FSIS Thermal Processing/ Commercially Sterile Self-Paced Training Course (2021)***<br><br>***Thermally Processed Products FSA Tool VS3 (2020)***<br>*All official establishments that produce thermally processed, commercially sterile meat and poultry products must meet the*	***Reg. (EC) 853/2004, annex I, processed products:***<br>*"Meat products" means processed products resulting from the processing of meat or from the further processing of such processed products, so that the cut surface shows that the product no longer has the characteristics of fresh meat.*	***EFSA - Opinion of the Scientific Panel on Biological Hazards on the request from the Commission related to Clostridium spp. in foodstuffs. 4.4.1Effect of heating.***<br>*Low acid canned foods are heated at 121°C for ca 3 min to eliminate spores of C. botulinum (12D reduction of Group I spores).*<br>***Introduction***<br>*Anaerobic spore-forming bacteria have been reported to spoil food products andto cause foodborne disease. Spoilage of dairy products, meat products, poultry products, fresh and canned fruits and vegetables, is caused by Clostridium barati, C. beijerinckii, C. bifermentans, C. butyricum, C. pasteurianum, C. puniceum, C.putrefaciens, C. sporogenes and C. tyrobutyricum (de Jong, 1989; Chapman,2001). They typically produce gas and/or putrid odours*	***YES***<br>*(the establishment must include in the process schedule all critical factors that could compromise the proper application of the thermal treatment).*<br><br>***Note:** When an establishment chooses to control biological hazards in its HACCP plan, thereby not following 9 CFR 431, the process schedule becomes the primary supporting document for the development of the HACCP plan itself. This schedule, developed or determined by a Processing Authority (PA), must include all critical factors that could compromise the proper application of the thermal treatment and, consequently, the safety and stability of the product. Since the microbiological hazards reasonably likely to occur in low-acid products are C. botulinum and C. sporogenes, the thermal treatment must ensure a 12-log reduction for C. botulinum and a 5-log reduction for C. sporogenes, as defined by the "**Microbiology of Thermally Processed***

	requirements in 9 CFR part 431. However, the establishment may choose to control biological hazards identified in the hazard analysis as reasonably likely to occur via the HACCP plan or via the controls in 9 CFR part 431 (see 9 CFR 417.2 (b)(3)).<br>*When an establishment chooses to control biological hazards in its HACCP plan, the process schedule*** developed by the processing authority typically becomes the primary supporting document for the development of the HACCP plan. These two documents should be consistent with each other. It is also common to see establishments use retort manufacturer's instructions and the canning regulations as supporting documentation for their decisions.*<br><br>****** 9 CFR 431 Definitions***<br>*Process schedule: the thermal process and any specified critical factors for a given canned product required to achieve shelf stability.*<br>*Thermal process: The heat treatment necessary to achieve shelf stability as determined by the establishment's processing authority. It is quantified in terms of: (1) Time(s) and temperature(s); or (2) Minimum product temperature.*<br>*Processing authority: The person(s) or organization(s) having expert knowledge of thermal processing requirements for foods in hermetically sealed containers,*			***Commercially Sterile and Shelf-Stable Meat and Poultry Products (2005)"****.*<br>*The development or determination of the process schedule can be supported by incubation tests, laboratory microbial testing results, PA's evaluation, and other supporting documentation such as retort manufacturer's instructions and the canning regulations.*<br>*In the event of modifications to ingredients, formulation, or treatments that could affect heat penetration or sterilization value, the PA must assess their impact. If such modifications compromise the effectiveness of the thermal treatment, the process schedule must be updated accordingly.*

	having access to facilities for making such determinations, and designated by the establishment to perform certain functions as indicated in this part.<br><br>***FSIS Directive 7530.2 III. background***<br>*…A process schedule developed by a Processing Authority is a supporting document.*<br><br>***9 CFR 431.3 Thermal processing***<br>*(b) Source of process schedules:*<br>*(1) Process schedules used by an establishment must be developed or determined by a processing authority.*<br>*(2) Any change in product formulation, ingredients, or treatments that are not already incorporated in a process schedule and that may adversely affect either the product heat penetration profile or sterilization value requirements must be evaluated by the establishment's processing authority. If it is determined that any such change adversely affects the adequacy of the process schedule, the processing authority must amend the process schedule accordingly.*<br>*(3) Complete records concerning all aspects of the development or determination of a process schedule, including any associated incubation tests, must be made available by the establishment to the Program employee upon request.*			

	Program employee: Any inspector or other individual employed by the Department or any cooperating agency who is authorized by the Secretary to do any work or perform any duty in connection with the Program.<br><br>***FSIS Directive 7530.1 VII Abnormal containers identified at an official FSIS inspected establishment***<br>*…any supporting documentation provided by the establishment, such as the incubation records, laboratory microbial testing results, the PA's evaluation, and other supporting documentation.*<br><br>***9 CFR 431.4 Critical factors and the application of the process schedule***			
**c) The contents of the HACCP plan**<br>*The HACCP plan shall, at a minimum:*<br>***1.*** *list the food safety hazards identified in accordance with paragraph (a) of this section, which must be controlled for each process.*	***FSIS-GD-2020-0008***<br><br>***Attachment 4: Hazard Analysis and Preventive Measures.***<br><br>***Attachment 5: CCP Determination.***	***Reg. (EC) 852/2004, Article 5, Paragraph 2:***<br>*The HACCP principles referred to in paragraph 1 consist of the following:*<br>***a.*** *identifying any hazards that must be prevented, eliminated or reduced to acceptable levels;*	***Commission Notice 2022/C 355/01***<br>*Appendix 4 Example of a decision tree to identify critical control points (CCP).*	***NO***<br>***Note:****. the decision tree proposed by Commission Notice 2022/C 355/01 traces the scheme set by Codex Alimentarius (Codex Alimentarius CXC 1-1969), US guideline "FSIS-GD-2020-0008" presents a similar, though not fully overlapping, scheme. Despite this, both schemes are fundamentally equivalent.*
2. *list the critical control points for each of the identified food safety hazards, including, as appropriate:*<br>***(i)*** *critical control points designed to control food safety hazards that could be introduced in the establishment, and*<br>***(ii)*** *critical control points designed to control food safety*	*//*	***b.*** *identifying the critical control points at the step or steps at which control is essential to prevent or eliminate a hazard or to reduce it to acceptable levels;*	*//*	***NO***

hazards introduced outside the establishment, including food safety hazards that occur before, during, and after entry into the establishment;				
3. *List the critical limits that must be met at each of the critical control points. Critical limits shall, at a minimum, be designed to ensure that applicable targets or performance standards established by FSIS, and any other requirement set forth in this chapter pertaining to the specific process or product, are met;*	//	***c.*** *establishing critical limits at critical control points which separate acceptability from unacceptability for the prevention, elimination or reduction of identified hazards;*	//	***NO***
4. *List the procedures, and the frequency with which those procedures will be performed, that will be used to monitor each of the critical control points to ensure compliance with the critical limits;*	//	***d.*** *establishing and implementing effective monitoring procedures at critical control points;*	//	***NO***
5. *Include all corrective actions that have been developed in accordance with 417.3(a) of this part, to be followed in response to any deviation from a critical limit at a critical control point; and*	//	***e.*** *establishing corrective actions when monitoring indicates that a critical control point is not under control;*	//	***NO***
6. *Provide for a recordkeeping system that documents the monitoring of the critical control points. The records shall contain the actual values and observations obtained during monitoring.*	//	*See letter **g***	//	***NO***
7. *List the verification procedures, and the frequency with which those procedures will be performed, that the establishment will use in*	//	***f.*** *establishing procedures, which shall be carried out regularly, to verify that the measures outlined in subparagraphs (a) to (e) are working effectively; and*	//	***NO***

accordance with 417.4 of this part.		***g.*** *establishing documents and records commensurate with the nature and size of the food business to demonstrate the effective application of the measures outlined in subparagraphs (a) to (f).*		
**d)** ***Signing and dating the HACCP plan***<br><br>*1. The HACCP plan shall be signed and dated by the responsible establishment individual. This signature shall signify that the establishment accepts and will implement the HACCP plan.*<br><br>*2. The HACCP plan shall be dated and signed:*<br><br>*3. (i) upon initial acceptance; (ii) upon any modification; and (iii) at least annually, upon reassessment, as required under 417.4(a)(3) of this part.*	//	//	***Commission Notice 2022/C 355/01***<br>***11. DOCUMENTATION AND RECORD KEEPING***<br>*Documents should be reviewed and signed and any deviation should be recorded and followed up by the person responsible for HACCP in the business.*	***NO***
**e)** *Pursuant to 21 U.S.C. 456, 463, 608, and 621, the failure of an establishment to develop and implement a HACCP plan that complies with this section, or to operate in accordance with the requirements of this part, may render the products produced under those conditions adulterated.*	//	***Reg. (EU) 625/2017, Article 138***<br>*Actions in the event of established non-compliance:*<br>*1. Where the non-compliance is established, the competent authorities shall take:*<br>*a) any action necessary to determine the origin and extent of the non-compliance and to establish the operator's responsibilities; and*<br>*b) appropriate measures to ensure that the operator concerned remedies the non-compliance and prevents further occurrences of such non-compliance.*<br>*When deciding which measures to take, the competent authorities shall take account of the nature of that*	//	***NO***

		non-compliance and the operator's past record with regard to compliance.<br>*2. When acting in accordance with paragraph 1 of this Article, competent authorities shall take any measure they deem appropriate to ensure compliance with the rules referred to in Article 1(2).*		
**417.3 *CORRECTIVE ACTIONS***	***FSIS-GD-2020-0008***	***Reg. (EC) 852/2004***	***Commission Notice 2022/C 355/01***	
**a)** *The written HACCP plan shall identify the corrective action to be followed in response to a deviation from a critical limit. The HACCP plan shall describe the corrective action to be taken, and assign responsibility for taking corrective action, to ensure:*<br>*1. the cause of the deviation is identified and eliminated;*<br>*2. the CCP will be under control after the corrective action is taken;*<br>*3. measures to prevent recurrence are established; and*<br>*4. no product that is injurious to health or otherwise adulterated as a result of the deviation enters commerce.*	***FSIS-GD-2020-0008***<br>*The optional worksheet in Attachment 6 can help the HACCP team make sure they have developed appropriate corrective actions for each CCP. Employees responsible for monitoring and other functions of the HACCP plan should be identified by title and not by name. This allows for appropriate substitutions when those individuals are not available.*	***Reg. (EC) 852/2004, Article 5, Paragraph 2:***<br>*The HACCP principles referred to in paragraph 1 consist of the following:*<br>*e) establishing corrective actions when monitoring indicates that a critical control point is not under control.*	***Annex II, 9. CORRECTIVE ACTIONS:***<br>*For each CCP, corrective actions should be planned in advance by the HACCP team, so that they can be taken without hesitation when monitoring indicates a deviation from the critical limit.*<br>*Such corrective actions should include:*<br>- *identification of the person(s) responsible for the implementation of the corrective action;*<br>- *means and action required to correct the observed deviation in the process*<br>- *action(s) to be taken with regard to products that have been manufactured during the deviation;*<br>- *written record of measures taken indicating all relevant information (for example: date, time, type of action, actor and subsequent verification check);*<br>- *consideration of (long term) actions to avoid repetition of the deviation.*	***NO***
**b)** *If a deviation not covered by a specified corrective action occurs, or if another unforeseen hazard arises, the establishment shall:*<br>*1. segregate and hold the affected product, at least until the requirements of paragraphs (b)(2) and (b)(3) of this section are met;*<br>*2. perform a review to determine the acceptability*	*//*	*//*	***Annex II, 9. CORRECTIVE ACTIONS:***<br>*For each CCP, corrective actions should be planned in advance by the HACCP team, so that they can be taken without hesitation when monitoring indicates a deviation from the critical limit.*<br>*Such corrective actions should include:*<br>- *identification of the person(s) responsible for the implementation of the corrective action;*<br>- *means and action required to correct the observed deviation in the process*	***NO***<br>***Note:*** *Although there is no specific reference in a regulatory text or European guideline to the cases in which a "deviation not covered by a specified corrective action occurs, or if another unforeseen hazard arises" it is believed that, by virtue of the general principle of food safety management, the food business operator is nevertheless required*

of the affected product for distribution;<br>3. *Take action, when necessary, with respect to the affected product to ensure that no product that is injurious to health or otherwise adulterated, as a result of the deviation, enters commerce;*<br>4. *Perform or obtain reassessment by an individual trained in accordance with 417.7 of this part, to determine whether the newly identified deviation or other unforeseen hazard should be incorporated into the HACCP plan.*			- *action(s) to be taken with regard to products that have been manufactured during the deviation;*<br>- *written record of measures taken indicating all relevant information (for example: date, time, type of action, actor and subsequent verification check);*<br>*consideration of (long term) actions to avoid repetition of the deviation.*	*to take appropriate corrective action. Such situations, in fact, fall squarely within the concept of deviation from compliance and, as such, require the implementation of corrective actions proportionate to the nature and severity of the nonconformity found, in compliance with the general obligation to ensure food safety*
**c)** *All corrective actions taken in accordance with this section shall be documented in records that are subject to verification in accordance with 417.4(a)(2)(iii) and the recordkeeping requirements of 417.5 of this part.*	*//*	*//*	***Annex II, 10. VALIDATION AND VERIFICATION PROCEDURES:***<br>*The HACCP team should establish verification procedures to confirm that the HACCP-based procedures are working correctly. Methods for verification may include:*<br>*- internal audits of HACCP-based procedures and their records;*<br>*- review of deviations and product dispositions; corrective actions taken with regard to the product.*<br>***Annex II, 11. DOCUMENTATION AND RECORD KEEPING:***<br>*Efficient and accurate record keeping is essential to the application of HACCP-based procedures. HACCP-based procedures should be documented in the HACCP-plan and continuously supplemented by records on findings.*<br>*Record examples are:*<br>- *outcome of monitoring activities on control measures;*<br>- *observed deviations and executed corrective actions;*<br>- *outcome of verification activities.*	***NO***

417.4 *VALIDATION, VERIFICATION, REASSESSMENT*	*FSIS-GD-2020-0008*	*Reg. (EC) 852/2004*	*Commission Notice 2022/C 355/01*	
**a)** *Every establishment shall validate the HACCP plan's adequacy in controlling the food safety hazards identified during the hazard analysis, and shall verify that the plan is being effectively implemented.*<br>*1. **Initial validation:***<br>*Upon completion of the hazard analysis and development of the HACCP plan, the establishment shall conduct activities designed to determine that the HACCP plan is functioning as intended. During this HACCP plan validation period, the establishment shall repeatedly test the adequacy of the CCP's, critical limits, monitoring and recordkeeping procedures, and corrective actions set forth in the HACCP plan. Validation also encompasses reviews of the records themselves, routinely generated by the HACCP system, in the context of other validation activities.*	*The FSIS Compliance Guideline HACCP Systems Validation April 2015 describes the process as two steps:*<br>*1) Design and 2) Execution.*<br>***Design** of the HACCP plan is the scientific or technical support for the HACCP system design – that is, the theoretical principles, expert advice from processing authorities, scientific or technical data, peer-reviewed journal articles, pathogen modeling programs, or other information demonstrating that particular process control measures can adequately prevent, reduce, or eliminate specific hazards.*<br>***Execution** of the HACCP plan is the in-plant validation data or the in-plant observations, measurements, microbiological test results, or other information demonstrating the control measures in the HACCP system can perform as expected within a particular establishment to achieve the intended food safety objective. These supporting validation documents are critical to the success of the HACCP plan and must be kept for the life of the plan.*<br>*•Frequency: Once over a period of the first 90 days of the new or revised HACCP System.*	//	*At the start of a new process or in case of a change to an existing process that is likely to affect food safety, the HACCP team should carry out validation activities, in particular gather evidence to confirm the capability of all elements of the HACCP plan, even if not explicitly mentioned in Article 5 of Regulation (EC) No 852/2004. Such evidence includes scientific publications, in-house testing (sampling and testing to see if biological and chemical hazards are under control), predictive microbiology, guidance developed by competent authorities, … demonstrating that the critical limits set, will result in the intended effect on the hazard (no growth, reduction, …).*	***NO***
2. Ongoing verification activities. *Ongoing verification activities include, but are not limited to:*	***Ongoing verification** ensures that the HACCP plan is working effectively on a day-to-*	//	*After the procedures based on the HACCP principles have been implemented, the HACCP team should establish verification procedures to confirm that the HACCP-based*	***YES***<br>***Note:** European legislation does not specify the ongoing verification methods However, the Commission Notice recommends*

(i) *the calibration of process-monitoring instruments;*<br>***(ii)*** *direct observations of monitoring activities and corrective actions; and*<br>***(iii)*** *the review of records generated and maintained in accordance with 417.5(a)(3) of this part.*	*day basis after initial validation is completed. This type of verification includes such tasks as calibrating monitoring instruments, observing monitoring activities and corrective actions, reviewing HACCP records to see that they are being made and kept according to the plan, the monitoring of critical limits and parameters of prerequisite programs to ensure that the critical operational parameters in the scientific support continue to be met, and testing for appropriate pathogens or other microorganisms.*<br>*If you rely on data from your prerequisite programs as a basis for determining that an identified hazard is not reasonably likely to occur, then the prerequisite program itself must be validated and you must maintain records that the programs are consistently and continuously implemented as designed.*<br>*•Frequency: Ongoing following completion of initial validation (i.e., day 91) and onward*		*procedures are working correctly. Methods for verification may include: random sampling and analysis, reinforced analysis or tests at selected critical points (intensified analysis of intermediate or end products e.g. on compliance with microbiological criteria, process hygiene criteria, Time/Temperature hazard reduction/elimination: follow up of relevant pathogens in heat-treated food products e.g. absence of Listeria monocytogenes, Salmonella etc…); internal audits of HACCP-based procedures and their records; inspection of operations (people compliance); confirmation that CCP monitoring is implemented and maintained by:*<br>*- control of the procedures/instructions;*<br>*- physical check on the process being monitored;*<br>*- verifying the calibration of instruments used for monitoring;*<br>*- verification of records (frequency, outcome of measuring results over period of time).*<br>*The frequency of verification should be sufficient to confirm that HACCP-based procedures are working effectively. The frequency of verification shall depend on the characteristics of the business (output, number of employees, nature of the food handled), the monitoring frequency, the accuracies of the employees, the number of deviations detected over time and the hazards involved. When the verification detects failures in the HACCP system, review of the system must be carried out. Verification should be carried out by someone other than the person who is responsible for performing the monitoring and corrective actions. Where certain verification activities cannot be performed in house, verification should be performed on*	*its implementation. In fact, internal audits and other systems of verification of the implemented HACCP plan are provided for. In addition, it is specified that these audits should be carried out by a person other than the person responsible for monitoring and implementing corrective actions.*

			behalf of the business by external experts or qualified third parties.	
3. (i) Reassessment of the HACCP plan. *Every establishment shall reassess the adequacy of the HACCP plan at least annually and whenever any changes occur that could affect the hazard analysis or alter the HACCP plan. Such changes may include, but are not limited to, changes in: raw materials or source of raw materials; product formulation; slaughter or processing methods or systems; production volume; personnel; packaging; finished product distribution systems; or, the intended use or consumers of the finished product. The reassessment shall be performed by an individual trained in accordance with 417.7 of this part. The HACCP plan shall be modified immediately whenever a reassessment reveals that the plan no longer meets the requirements of 417.2(c) of this part.*<br>***(ii)*** *Each establishment must make a record of each reassessment required by paragraph (a)(3)(i) of this section and must document the reasons for any changes to the HACCP plan based on the reassessment, or the reasons for not changing the HACCP plan based on the reassessment. For annual reassessments, if the establishment determines that no changes are needed to its HACCP plan, it is not required to*	*Reassessment is an overall review of the plan that must be performed at least annually, whenever any changes occur that could affect the hazard analysis or alter the HACCP plan and in response to a deviation not covered by a specific corrective action (unforeseen hazard). Reassessment is similar to validation in that it considers, in general, whether the plan is adequate, rather than focusing on the plan's daily operations.*<br>*•Frequency: Annually and whenever changes occur that affect the hazard analysis or HACCP plan.*	***Reg. (EC) 852/2004, Article 5, paragraph 2:***<br>*When any modification is made in the product, process, or any step, food business operators shall review the procedure and make the necessary changes to it.*	*At the start of a new process or in case of a change to an existing process that is likely to affect food safety, the HACCP team should carry out validation activities, in particular gather evidence to confirm the capability of all elements of the HACCP plan.*<br>*Examples of changes that may require re-validation include:*<br>*- change in raw material or in product, processing conditions (factory layout and environment, process equipment, cleaning and disinfection programme);*<br>*- change in packaging, storage or distribution conditions;*<br>*- change in consumer use;*<br>*- receipt of any information on a new hazard associated with the product.*	***NO***

document the basis for this determination.				
b) Reassessment of the hazard analysis<br>*Any establishment that does not have a HACCP plan because a hazard analysis has revealed no food safety hazards that are reasonably likely to occur shall reassess the adequacy of the hazard analysis whenever a change occurs that could reasonably affect whether a food safety hazard exists. Such changes may include, but are not limited to, changes in: raw materials or source of raw materials; product formulation; slaughter or processing methods or systems; production volume; packaging; finished product distribution systems; or, the intended use or consumers of the finished product.*	//	***Reg. (EC) 852/2004, Article 5:***<br>*Food business operators shall put in place, implement and maintain a permanent procedure or procedures based on the HACCP principles…*<br>*When* ***any modification*** *is made in the product, process, or any step, food business operators shall review the procedure and make the necessary changes to it.*	*In case of a change to an existing process that is likely to affect food safety, the HACCP team should carry out validation activities, in particular gather evidence to confirm the capability of all elements of the HACCP plan.*<br>*Examples of changes that may require re-validation include:*<br>*- change in raw material or in product, processing conditions (factory layout and environment, process equipment, cleaning and disinfection programme);*<br>*- change in packaging, storage or distribution conditions;*<br>*- change in consumer use;*<br>*- receipt of any information on a new hazard associated with the product.*	***NO***<br>***Note:*** *Article 5 of Reg. (EC) 852/2004 states that food businesses operators must implement a system based on HACCP principles and that this system must be reviewed when there are significant changes in the "product, process, or any step." In practice this means that if an establishment does not have an HACCP plan because a hazard analysis has revealed no food safety hazards that are reasonably likely to occur, it must still review the adequacy of the hazard analysis whenever a change occurs that could reasonably affect whether a food safety hazard exists. For example, if there are changes in raw materials or source of raw materials; product formulation; slaughter or processing methods or systems; production volume, etc.*
417.5 RECORDS	***FSIS-GD-2020-0008***	***Reg. (EC) 852/2004***<br>***Reg. (EU) 2017/625***	***Commission Notice 2022/C 355/01***	
**a)** *The establishment shall maintain the following records documenting the establishment's HACCP plan:*<br>*1. the written hazard analysis prescribed in 417.2(a) of this part, including all supporting documentation;*<br>*2. the written HACCP plan, including decision making documents associated with the selection and development of CCP's and critical limits, and documents supporting both the monitoring and*	*Record-keeping is an essential feature of a HACCP system that must be planned and carried out as carefully as any other element. Keep records of both the development of your HACCP plan and the operation of the HACCP system.*	***III. Record-keeping***<br>*Food business operators are to keep and retain records relating to measures put in place to control hazards in an appropriate manner and for an appropriate period, commensurate with the nature and size of the food business. Food business operators are to make relevant information contained in these records available to the competent authority and receiving food business operators on request.*<br>***Article 5, Paragraph 2, letter g:***<br>*Establishing documents and records commensurate with the*	***11- Documentation and Record keeping***<br>*Efficient and accurate record keeping is essential to the application of HACCP-based procedures. HACCP-based procedures should be documented in the HACCP-plan and continuously supplemented by records on findings. Documentation and record keeping should be appropriate to the nature and size of the operation and sufficient to assist the business to verify that the HACCP-based procedures are in place and being maintained. Expert developed HACCP guidance materials (e.g. sector-specific HACCP guides) may be utilized as part of the documentation, provided*	***NO***

verification procedures selected and the frequency of those procedures.<br>3. *records documenting the monitoring of CCP's and their critical limits, including the recording of actual times, temperatures, or other quantifiable values, as prescribed in the establishment's HACCP plan; the calibration of process-monitoring instruments; corrective actions, including all actions taken in response to a deviation; verification procedures and results; product code(s), product name or identity, or slaughter production lot. Each of these records shall include the date the record was made.*		*nature and size of the food business to demonstrate the effective application of the measures outlined in subparagraphs (a) to (f).*	*that those materials reflect the specific food operations of the business. Documents should be reviewed and signed and any deviation should be recorded and followed up by the person responsible for HACCP in the business. Recommended documentation includes:*<br>*- GHP documentation, see Annex I, Section 6.*<br>*- description of the preparatory stages (before 7 principles);*<br>*- hazard analysis, including hazard identification;*<br>*- CCP (and OPRP) identification;*<br>*- critical limit (action criteria) determination;*<br>*- validation activities;*<br>*- corrective actions anticipated;*<br>*- description of planned monitoring and verification activities (what, who, when);*<br>*- record forms;*<br>*- modifications to the HACCP-based procedures;*<br>*- supporting documents (generic guides, scientific evidence, etc.).*	
**b)** *Each entry on a record maintained under the HACCP plan shall be made at the time the specific event occurs and include the date and time recorded, and shall be signed or initialed by the establishment employee making the entry.*	*The best recordkeeping system is usually the simplest one that you can easily integrate into an existing operation; consider using simple, understandable forms that will work well in your situation. Make sure your employees know exactly what is expected if they are responsible for making a record entry: they must sign/initial, timestamp, and date the records at the time the specific event occurs (9 CFR 417.5(b)). Also consider the best place to store the records.*	*//*	*//*	***NO***
**c)** *Prior to shipping product, the establishment shall review the records associated with the production of that product, documented in accordance with*	*That is commonly referred to as pre-shipment review. It can assure that you have done everything in your HACCP system before you ship a*	*//*	*//*	***YES***<br><br>***Note:*** *European legislation does not require pre-shipment review.*

this section, to ensure completeness, including the determination that all critical limits were met and, if appropriate, corrective actions were taken, including the proper disposition of product. Where practicable, this review shall be conducted, dated, and signed by an individual who did not produce the record(s), preferably by someone trained in accordance with 417.7 of this part, or the responsible establishment official.	*product. A completed pre-shipment review indicates that the product is free from food safety hazards, as well as other causes of adulteration and is ready for commerce.*<br>*Sign the review only after someone has reviewed all lot specific documentation and all establishment and FSIS testing for adulterants such as E. coli O157:H7, drug residues, etc., are completed and negative. That includes CCP monitoring and verification but may also include testing results in a prerequisite program, letters of guarantee, or other records associated with the production of that lot.*			
d) Records maintained on computers.<br>*The use of records maintained on computers is acceptable, provided that appropriate controls are implemented to ensure the integrity of the electronic data and signatures.*	*//*	*//*	***11- Documentation and Record keeping***<br>*Records should be kept for an appropriate period of time in any format. That period should be long enough to ensure information to be available in case of an alert that can be traced back to the food in question.*	***NO***
e) Record retention<br>*1. Establishments shall retain all records required by paragraph (a)(3) of this section as follows: for slaughter activities for at least one year; for refrigerated product, for at least one year; for frozen, preserved, or shelf-stable products, for at least two years.*<br>*2. Off-site storage of records required by paragraph (a)(3) of this section is permitted after six months, if such records can be retrieved and provided, on-*	*//*	***Article 5, paragraph 4:***<br>*Food business operators shall:*<br>***c)*** *retain any other documents and records for an appropriate period.*<br><br>***III. Record keeping:***<br>*Food business operators are to keep and retain records relating to measures put in place to control hazards in an appropriate manner and for an appropriate period, commensurate with the nature and size of the food business. Food business operators are to make relevant information contained in these records available to the competent authority and receiving food business operators on request.*	***11- Documentation and Record keeping***<br>*Records should be kept for an appropriate period of time in any format. That period should be long enough to ensure information to be available in case of an alert that can be traced back to the food in question.*	***NO***<br><br>***Note:*** *European legislation do not define a mandatory minimum time period. However, it establishes that they must be kept for an appropriate period and made available to the competent authority when requested. Therefore, we can say that the US and EU provisions on this point are equivalent.*

site, within 24 hours of an FSIS employee's request.				
f) Official review<br>*All records required by this part and all plans and procedures required by this part shall be available for official review and copying.*	//	***Reg. (EC) 852/2004***<br>***III. Record keeping:***<br>*Food business operators are to keep and retain records relating to measures put in place to control hazards in an appropriate manner and for an appropriate period, commensurate with the nature and size of the food business. Food business operators are to make relevant information contained in these records available to the competent authority and receiving food business operators on request*<br>***Reg. (EU) 2017/625, Article 15***<br>***Obligations of operators:***<br>*1. To the extent that this is necessary for the performance of official controls or of other official activities, operators shall, where required by the competent authorities, give staff of the competent authorities access to:*<br>*a) the equipment, means of transport, premises and other places under their control and their surroundings;*<br>*b) their computerized information management systems;*<br>*c) the animals and goods under their control;*<br>*d) their documents and any other relevant information.*<br>*2. During official controls and other official activities, operators shall assist and cooperate with the staff of the competent authorities and organic control authorities in the accomplishment of their tasks.*<br>*6. The obligations of operators set out in this Article shall also apply in cases where official controls and other official activities are performed by official veterinarians,*	//	***NO***

		official plant health officers, delegated bodies, control authorities and natural persons to which certain official control tasks or certain tasks related to other official activities have been delegated.		
417.6 INADEQUATE HACCP SYSTEM				
A HACCP system may be found to be inadequate if:<br>*a. the HACCP plan in operation does not meet the requirements set forth in this part;*<br>*b. establishment personnel are not performing tasks specified in the HACCP plan;*<br>*c. the establishment fails to take corrective actions, as required by 417.3 of this part;*<br>*d. HACCP records are not being maintained as required in 417.5 of this part; or*<br>*e. adulterated product is produced or shipped.*	//	//	//	***NO***<br>***Note:*** *Reg. (EC) 852/2004 does not provide an explicit definition of the characteristics of an "inadequate" HACCP system; however, it does establish the essential requirements that an HACCP system must meet in order for it to be effective in managing food safety. It follows that failure to meet these requirements effectively results in the HACCP being inadequate, as it does not comply with regulatory requirements and is potentially ineffective in ensuring hazard control and food safety.*
417.7 TRAINING	***FSIS-GD-2020-0008***	***Reg (EC) 852/2004***<br>***Reg. (UE) 2021/382***	***Commission Notice 2022/C 355/01***	
**a)** *Only an individual who has met the requirements of paragraph (b) of this section, but who need not be an employee of the establishment, shall be permitted to perform the following functions:*<br>*1. development of the HACCP plan, in accordance with 417.2(b) of this part, which could include adapting a generic model that is appropriate*	//	***Reg. (EC) 852/2004, chapter XII,*** *Food business operators are to ensure:*<br>*1. that food handlers are supervised and instructed and/or trained in food hygiene matters commensurate with their work activity;*<br>*2. that those responsible for the development and maintenance of the procedure referred to in Article 5(1) of this Regulation or for the operation of relevant guides have received adequate*	***8. Training***<br>*Staff of FBOs should be supervised and instructed and/or trained in food hygiene matters appropriate to their role, and those responsible for developing and maintaining the food safety management system should be suitably trained in the application of GHP, other PRP and HACCP–based procedures.*<br>*The management shall make sure that staff taking part in the relevant processes demonstrate sufficient skills and are aware of the hazards identified (if any) and of the critical points in the*	***NO***

for the specific product; and<br>2. *reassessment and modification of the HACCP plan, in accordance with 417.3 of this part.*		*training in the application of the HACCP principles; and*<br>3. *compliance with any requirements of national law concerning training programmes for persons working in certain food sectors.*<br>***Reg. (EU) 2021/382 (amending the Annexes to Regulation (EC) 852/2004), Chapter XIa ,***<br>*Food Safety Culture*<br>***1.*** *Food business operators shall establish, maintain and provide evidence of an appropriate food safety culture by fulfilling the following requirements:*<br>*a) commitment of the management, in accordance with point 2, and all employees to the safe production and distribution of food;*<br>*b) leadership towards the production of safe food and to engage all employees in food safety practices;*<br>*c) awareness of food safety hazards and of the importance of food safety and hygiene by all employees in the business;*<br>*d) open and clear communication between all employees in the business, within an activity and between consecutive activities, including communication of deviations and expectations;*<br>*e) availability of sufficient resources to ensure the safe and hygienic handling of food.*<br>***2.*** *Management commitment shall include:*<br>*a) ensuring that roles and responsibilities are clearly communicated within each activity of the food business;*	*production, storage, transport and/or distribution process. They must also show awareness of the corrective measures, the preventive measures and monitoring and recording procedures applicable in the business, in accordance with Chapter XII of Annex II to Regulation (EC) 852/2004. A distinction should be made between training on hygiene in general (all employees) and specific HACCP training. The employees who monitor/manage or verify critical control points (CCP) should be trained in the procedures based on the HACCP principles appropriate to their tasks. Possible refresher training and its frequency should be considered according to the needs of the establishment and demonstrated skills. Training as referred to in Chapter XII of Annex II to Regulation (EC) 852/2004 must be seen in a broad context. In such context, appropriate training does not necessarily involve participation in formal training courses. Skills and knowledge can also be achieved through access to technical information and advice from professional organizations or from the competent authorities, suitable on-the-job/in house training, and guides to good practice etc. GHP, other PRP and HACCP training of staff in food businesses should be proportionate to the size and the nature of the business and take into account specific risks related to the nature of the activity. The importance of training has been enhanced due to the introduction of the (mandatory) requirement for a food safety culture in Regulation (EC) 852/2004 in March 2021. Training will often be the most important tool to achieve a good food safety culture or to serve as corrective action in case shortcomings are detected when evaluating the extent of the food safety culture.*	

		b) maintaining the integrity of the food hygiene system when changes are planned and implemented;<br>*c) verifying that controls are being performed timely and efficiently and documentation is up to date;*<br>*d) ensuring that the appropriate training and supervision are in place for personnel;*<br>*e) ensuring compliance with relevant regulatory requirements;*<br>*f) encouraging continual improvement of the food safety management system of the business, where appropriate, taking into account developments in science, technology and best practices.*<br>*3. The implementation of the food safety culture shall take account of the nature and size of the food business.*		
**b)** *The individual performing the functions listed in paragraph (a) of this section shall have successfully completed a course of instruction in the application of the seven HACCP principles to meat, poultry, or egg products processing, including a segment on the development of a HACCP plan for a specific product and on record review.*	***FSIS-GD-2020-0008***<br>*One resource you must include is someone trained in HACCP in accordance with the requirements of 9 CFR 417.7(b). This individual does not need to be a company employee but does need to be available to you for HACCP plan development and certain other functions such as reassessing your HACCP plans. FSIS does not certify or provide HACCP training for establishment employees. You may obtain HACCP training that includes the seven HACCP principles online or through local university extension offices or trade organizations.*	***Reg. (EC) 852/2004, recital n. 13:***<br>*Successful implementation of the procedures based on the HACCP principles will require the full cooperation and commitment of food business employees. To this end, employees should undergo training. The HACCP system is an instrument to help food business operators attain a higher standard of food safety. The HACCP system should not be regarded as a method of self-regulation and should not replace official controls.*<br>***Reg. (EC) 852/2004, chapter XII:***<br>*Food business operators are to ensure:*<br>*1. that food handlers are supervised and instructed and/or trained in food hygiene matters commensurate with their work activity;*<br>*2. that those responsible for the development and maintenance of the procedure referred to in Article*	*//*	***NO***

		5(1) of this Regulation or for the operation of relevant guides have received adequate training in the application of the HACCP principles; and<br>*3. compliance with any requirements of national law concerning training programmes for persons working in certain food sectors.*		
**417.8 AGENCY VERIFICATION**		***Reg (EU) 2017/625,***<br>***Reg. (EU) 2019/627***		
FSIS will verify the adequacy of the HACCP plan(s) by determining that each HACCP plan meets the requirements of this part and all other applicable regulations. Such verification may include:<br>*a. Reviewing the HACCP plan;*<br>*b. Reviewing the CCP records;*<br>*c. Reviewing and determining the adequacy of corrective actions taken when a deviation occurs;*<br>*d. Reviewing the critical limits;*<br>*e. Reviewing other records pertaining to the HACCP plan or system;*<br>*f. Direct observation or measurement at a CCP;*<br>*g. Sample collection and analysis to determine the product meets all safety standards; and*<br>*h. On-site observations and record review.*	*//*	***Reg (EU) 2017/625, Article 14 Methods and techniques for official controls****:*<br>*Official control methods and techniques shall include the following as appropriate:*<br>*d) an assessment of procedures on good manufacturing practices, good hygiene practices, good farming practices, and of procedures based on the principles of hazard analysis critical control points (HACCP).*<br>***Reg. (EU) 2019/627, recital n. 13:***<br>*Specific requirements for auditing by the competent authorities should also be maintained to ensure the uniform practical verification of compliance with Union requirements on products of animal origin. Auditing is of particular interest for the verification of general and specific hygiene requirements and the application of procedures based on hazard analysis and critical control points (HACCP).*<br>***Reg. (EU) 2019/627, Title II, chapter I, article 3, paragraph 2:***<br>*When auditing procedures based on hazard analysis and critical control points (HACCP), as laid down in Article 5 of Regulation (EC) No 852/2004, the competent authorities*	*//*	***NO***

		shall verify that food business operators handling products of animal origin apply such procedures continuously and properly.		

Table S2. Product categorization according to 9 CFR 417(b)(1).

Food processing categories	Finished products			
	Raw product	NRTE product	RTE product	TPCS product
Slaughter				
Raw product - ground				
Raw product - not ground				
Thermally processed commercially sterile				✓
Not heat treated - shelf stable				
Heat treated - shelf stable				
Fully cooked - not shelf stable				
Heat treated but not fully cooked - not shelf stable				
Product with secondary inhibitors - not shelf stable				

NRTE, not ready to eat; RTE, ready to eat; TPCS, thermally processed commercially sterile.
